# Characterizing CO and NO_*y*_ Sources and Relative Ambient Ratios in the Baltimore Area Using Ambient Measurements and Source Attribution Modeling

**DOI:** 10.1002/2017jd027688

**Published:** 2018-03-27

**Authors:** Heather Simon, Luke C. Valin, Kirk R. Baker, Barron H. Henderson, James H. Crawford, Sally E. Pusede, James T. Kelly, Kristen M. Foley, R. Chris Owen, Ronald C. Cohen, Brian Timin, Andrew J. Weinheimer, Norm Possiel, Chris Misenis, Glenn S. Diskin, Alan Fried

**Affiliations:** 1 Office of Air Quality Planning and Standards, U.S. Environmental Protection Agency, Research Triangle Park, NC, USA,; 2 National Exposure Research Laboratory, U.S. Environmental Protection Agency, Research Triangle Park, NC, USA,; 3 NASA Langley Research Center, Hampton, VA, USA,; 4 Department of Environmental Sciences, University of Virginia, Charlottesville, VA, USA,; 5 Department of Chemistry, University of California, Berkeley, CA, USA,; 6 Department of Earth and Planetary Science, University of California, Berkeley, CA, USA,; 7 National Center for Atmospheric Research, Boulder, CO, USA,; 8 Institute of Arctic and Alpine Research, University of Colorado Boulder, Boulder, CO, USA

## Abstract

Modeled source attribution information from the Community Multiscale Air Quality model was coupled with ambient data from the 2011 Deriving Information on Surface conditions from Column and Vertically Resolved Observations Relevant to Air Quality Baltimore field study. We assess source contributions and evaluate the utility of using aircraft measured CO and NO_*y*_ relationships to constrain emission inventories. We derive ambient and modeled ΔCO:ΔNO_*y*_ ratios that have previously been interpreted to represent CO:NO_*y*_ ratios in emissions from local sources. Modeled and measured ΔCO:ΔNO_*y*_ are similar; however, measured ΔCO:ΔNO_*y*_ has much more daily variability than modeled values. Sector-based tagging shows that regional transport, on-road gasoline vehicles, and nonroad equipment are the major contributors to modeled CO mixing ratios in the Baltimore area. In addition to those sources, on-road diesel vehicles, soil emissions, and power plants also contribute substantially to modeled NO_*y*_ in the area. The sector mix is important because emitted CO:NO_*x*_ ratios vary by several orders of magnitude among the emission sources. The model-predicted gasoline/diesel split remains constant across all measurement locations in this study. Comparison of ΔCO:ΔNO_*y*_ to emitted CO:NO_*y*_ is challenged by ambient and modeled evidence that free tropospheric entrainment, and atmospheric processing elevates ambient ΔCO:ΔNO_*y*_ above emitted ratios. Specifically, modeled ΔCO:ΔNO_*y*_ from tagged mobile source emissions is enhanced 5–50% above the emitted ratios at times and locations of aircraft measurements. We also find a correlation between ambient formaldehyde concentrations and measured ΔCO:ΔNO_*y*_ suggesting that secondary CO formation plays a role in these elevated ratios. This analysis suggests that ambient urban daytime ΔCO:ΔNO_*y*_ values are not reflective of emitted ratios from individual sources.

## 1. Introduction

Identification of specific sources of precursor emissions to ozone (O_3_) is useful for air quality planning associated with the O_3_ National Ambient Air Quality Standards. Quantifying source contributions from distinct sources or groups of sources to secondarily formed pollutants such as O_3_ provides important information about which emission controls may be most effective to improve air quality at a given time and place. Information about trace gases obtained from intensive field campaigns provides an opportunity to better understand sources of O_3_ precursors (nitrogen oxides, NO_*x*_, and volatile organic compounds, VOCs) in specific areas, and how well they may be characterized in emission inventories and air quality models. A critical challenge is to maximize the strengths of field study information, recognize limitations, and supplement with additional sources of data to best characterize and quantify source-receptor relationships. This is especially challenging with secondarily formed pollutants such as O_3_ that results from complex chemical and physical processes in the atmosphere.

Source contribution to air quality has historically been estimated using methods either based on ambient measurements or model applications. One ambient-based approach for source attribution involves comparing a measured reactive tracer (e.g., oxidized nitrogen, sulfur dioxide, and particulate organic carbon) with another compound considered relatively inert on short time and spatial scales, usually carbon monoxide (CO), to remove uncertainties relating to atmospheric transport (Arriaga-Colina et al., 2004; Buhr et al., 1992; Fujita et al., 1992; Harley et al., 1997). The CO:NO_*x*_ or CO:NO_*y*_ ratio specifically was first used in the early 1990s. Fujita et al. (1992) compared measured morning ratios in the Los Angeles Basin to local and basin-wide emission ratios from on-road mobile sources alone versus emissions ratios from all sources to determine at which sites air pollution was dominated by on-road mobile sources. Buhr et al. (1992) and Buhr et al. (1995) used principal component analysis of CO, SO_2_, and NO_*y*_ to identify major factors influencing air pollution mixing ratios in rural Pennsylvania and rural Alabama. They then used comparisons of CO:NO_*y*_ and CO:SO_2_ ratios from each factor to ratios from known emission sources to determine which major emissions sources were represented by each factor that had been identified. Li et al. (1997) also used principal component analysis to identify air pollution factors in Vancouver Canada and then applied CO:NO_*x*_ ratios to identify the sources represented by each factor and their relative importance. Mellios et al. (2006) used measured CO:NO_*x*_ ratios in cities in five European countries to compare against on-road mobile source emissions modeling simulations done with varying proportions of urban driving to estimate what portion of the vehicle emissions came from those types of driving conditions. Finally, Guo et al. (2009) evaluated CO:NO_*x*_ ratios across monitoring sites in the Pearl River Delta and Hong Kong to determine which sites were influenced by local versus regional air pollution sources.

Source apportionment approaches implemented in photochemical transport models track pollutants emitted from specific sources through the transport and chemistry calculations within the model and provide information on estimated source contributions to pollutant mixing ratios at every time step and at each grid location within the modeling domain. Source apportionment implementations in photochemical grid models have been used to differentiate modeled contribution to primary and secondary pollutants from broad source sectors (Baker et al., 2016; Fann et al., 2013; Goldberg et al., 2016), specific facilities (Baker & Kelly, 2014; Baker & Woody, 2017), and lateral boundary chemical inflow using reactive tracers (Baker et al., 2015; Dolwick et al., 2015).

Source apportionment approaches implemented in air quality models can be limited by deficiencies in model inputs (e.g., emissions or meteorology) and formulation (e.g., chemistry and deposition) approaches (Kwok et al., 2013, 2015). Since modeled source contribution estimates rely on accurate emissions values from the sources being tracked, it is especially important to use ambient data to evaluate emission estimates where possible. The CO:NO_*y*_ ratio, in addition to being used to determine source strengths, has also been used in a multitude of studies to evaluate NO_*x*_ and/or CO emission estimates from on-road vehicles in Southern California (Fujita et al., 1992; Harley et al., 1997; Pierson et al., 1990), California’s San Joaquin Valley (Marr et al., 2002), Boise Idaho (Walace et al., 2012), Central Pennsylvania (Buhr et al., 1992), Baltimore (Anderson et al., 2014), Athens (Kourtidis et al., 1999), Mexico City (Arriaga-Colina et al., 2004), Sao Paulo (Vivanco & de Fatima Andrade, 2006), and across urban U.S. monitoring sites (Parrish, 2006). Various studies have used different techniques to isolate the signal from on-road vehicles from ambient measurements. Some have restricted the ambient measurements to locations expected to be dominated by vehicle emissions like traffic tunnels or street canyons (Kourtidis et al., 1999; Pierson et al., 1990; Walace et al., 2012). Others have restricted ambient measurement to urban environments in the early morning when vehicles are expected to be the major emission source (Arriaga-Colina et al., 2004; Fujita et al., 1992; Harley et al., 1997; Luke et al., 2010; Marr et al., 2002; Parrish, 2006; Vivanco & de Fatima Andrade, 2006). In this paper we explore some of the strengths and limitations of using ambient CO:NO_*y*_ measurements to evaluate emissions from a single source category when measurements are not made in a near-source environment.

One particular region that has been the focus of a recent intensive field campaign is the Baltimore metropolitan area, which has historically been in nonattainment of the O_3_ National Ambient Air Quality Standards. National Aeronautics and Space Administration’s (NASA) Deriving Information on Surface Conditions from Column and Vertically Resolved Observations Relevant to Air Quality (DISCOVER-AQ) field study over the Baltimore, Maryland region during July 2011 included intensive measurements at the surface, aloft, and over the Chesapeake Bay (Crawford et al., 2014; Crawford & Pickering, 2014). Data from this field campaign has been used to examine regional emissions, evaluate air quality models used for O_3_ planning purposes, and compare satellite with ground-based measurements to better understand the complex source-receptor relationships in the Baltimore area (Anderson et al., 2014; Flynn et al., 2014; Follette-Cook et al., 2015; Goldberg et al., 2016; Ott et al., 2016). The CO:NO_*y*_ ratio has been used to assess on-road mobile emissions in different parts of the Baltimore region using the July 2011 DISCOVER-AQ aircraft measurements of NO_*y*_ and CO (Anderson et al., 2014). That study found disagreements between ambient ratios and emission inventory-based ratios and used those findings to conclude that current mobile source emission models overpredict NO_*x*_ emissions by 50–70% but predict CO emissions within 20%.

In this work, we leverage the extensive DISCOVER-AQ ambient data set and augment it with fine-scale source apportionment photochemical modeling in which source contributions to CO, NO_*y*_, and O_3_ were tracked to further explore the utility of using CO:NO_*y*_ ratios from aircraft measurements for the purpose of characterizing local emission sources. We begin with a brief analysis of model performance to verify the model’s value in characterizing air pollution during this episode (section 3.1.1). We then provide model estimates of the major emission sources impacting CO, NO_*y*_, and O_3_ mixing ratios at different times and locations during the field campaign (section 3.1.2) to provide context in understanding ambient and modeled CO:NO_*y*_ ratios. Next we explore how this ratio varies across those same times and locations both in the ambient data and in the model (section 3.2). Finally, we explore how local emissions, entrainment of free tropospheric air, and atmospheric processing (i.e., aging) impact this ratio (section 3.3) and provide insight into the strengths and limitations of relying on ambient measurements of photochemically active tracers to evaluate emission inventories for specific sectors.

## 2. Methods

### 2.1. DISCOVER-AQ P3B Aircraft Measurements

**Figure 1.**
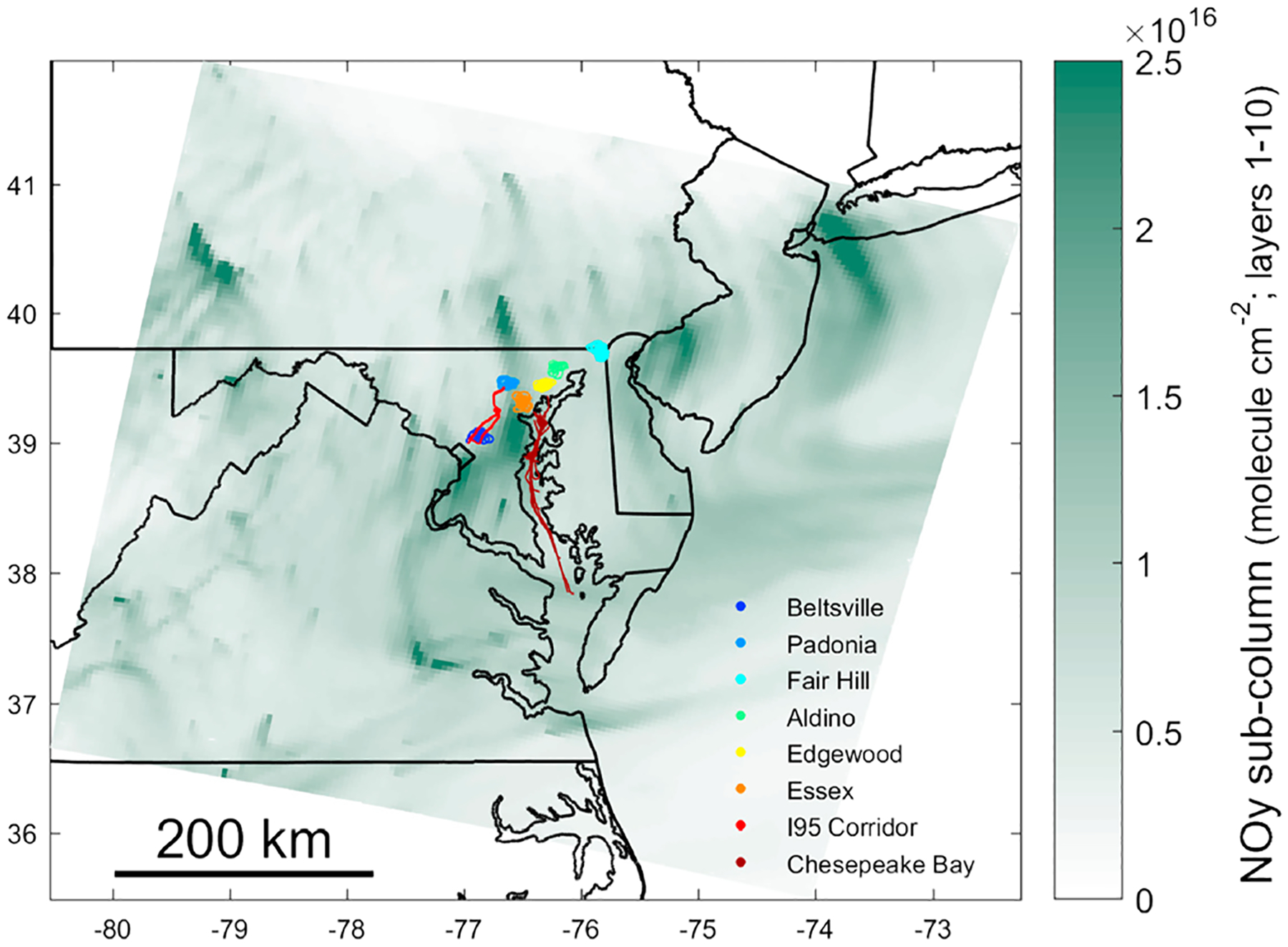
Flight tracks on all days tagged by location and simulated NO_*y*_ over the entire domain 27 July 2011 at 13 UTC, averaged over the lowest 10 model layers. “Onflight” locations when the plane traversed between sampling locations are not shown. The green shaded area shows the extent of the 4 km Community Multiscale Air Quality modeling domain and depicts an example of morning boundary layer NO_*y*_ concentration spatial gradients.

During the DISCOVER-AQ field campaign the NASA P-3B aircraft measured a suite of pollutants on 14 days in July 2011 in the Baltimore and Washington D.C. area. Flights times varied from day to day with take offs ranging from 5:30 a.m. to 2:15 p.m. and landing between 1 p.m. and 7 p.m. local time. Figure 1 provides a map of flight paths on all days, color coded by location tag.

A brief summary of measurements is given below. Additional details are provided in the supporting information and in the cited references. CO was measured on the NASA P-3B aircraft by the Differential Absorption CO Measurements instrument (Sachse et al., 1987). Formaldehyde (CH_2_O) was measured on the NASA P-3B aircraft using the Difference Frequency Generation Absorption Spectrometer instrument, comprehensive details for which can be found in Fried et al. (2016, and references therein).

NO, NO_2_, and NO_*y*_, and O_3_ were measured with the National Center for Atmospheric Research four-channel chemiluminescence instrument on board the P-3B aircraft (Ridley & Grahek, 1990; Ridley et al., 1994). NO_2_ was also measured by laser-induced fluorescence (LIF) (Thornton et al., 1999). Thermal dissociation (TD) is coupled with LIF to measure peroxy nitrates (PNs; ∑RO_2_NO_2_), alkyl nitrates (ANs; ∑RNO_2_), and nitric acid (HNO_3_) (Day et al., 2002). The TD-LIF instrument used here is a two-cell system. Data were collected at 4 Hz and averaged to 1 s, such that measurements were made in the following cycle: NO_2_ and PNs (8 s), ANs (8 s), NO_2_ + PNs (8 s), and HNO_3_ (8 s), with six offline seconds between each species sampling period.

Since measurements were available both for total NO_*y*_ and for the daytime NO_*y*_ components from the TD-LIF, we were able to compare mixing rations of NO_*y*_ using two methods: (1) total NO_*y*_ and (2) reconstructed NO_*y*_ (∑NO_*y*,*i*_ = NO + NO_2_ (measured by LIF) + PNs + ANs + HNO_3_). Measurements of all five-component species are needed to calculate ∑NO_*y*,*i*_. As discussed above, the TD-LIF instrument cycled between PN, AN, and HNO_3_ measurements, so to fill in values for the ∑NO_*y*,*i*_ calculation, missing values of each species were replaced with the last measured value so long as it was within 2.5 min of the missing value. While we allowed gap filling with measurements up to 2.5 min apart, most of the gap filling used measurements that were made within less than 1 min of the missing value. The aircraft traversed approximately 250 m vertically, on average, over a 1 min span during a spiral. Therefore, the ∑NO_*y*,*i*_ values do represent a less vertically resolved measurement than NO_*y*_ from the National Center for Atmospheric Research instrument that adds some uncertainty to this value. Even with this gap-filling method there are fewer data points for ∑NO_*y*,*i*_ than NO_*y*_ because of missing data.

An analysis in the supporting information shows that NO_*y*_ is consistently higher than ∑NO_*y*,*i*_ on five flight days and consistently lower than ∑NO_*y*,*i*_ on the other nine flight days. While there does not appear to be a systematic bias across all days, there were some times for which these two NO_*y*_ measurements differed substantially (see Figure S2 in the supporting information). In light of the uncertainty about the measurement differences at certain times between the NO_*y*_ and ∑NO_*y*,*i*_, we used both NO_*y*_ and ∑NO_*y*,*i*_ for this analysis.

### 2.2. Source Apportionment Modeling to Match the Period of the July 2011 DISCOVER-AQ Field Study

The model domain used a 4 km × 4 km grid resolution and covered the mid-Atlantic corridor shown in green in Figure 1. The modeled vertical atmosphere extends to 50 mb and is resolved with 35 layers that are thinnest nearest the surface to best resolve diurnal variability in the surface mixing layer. Outputs from the Weather Research and Forecasting model (Skamarock et al., 2008) version 3.7 were used as inputs to the Community Multiscale Air Quality (CMAQ) model (Byun & Schere, 2006) and for emissions processing. CMAQv5.0.1 was run using the CB05TUCl chemical mechanism (Sarwar et al., 2012; Whitten et al., 2010). This mechanism includes conventional isoprene chemistry but does not incorporate recent isoprene mechanism updates. Hourly chemical inflow is provided by an annual 2011 CMAQ simulation covering the contiguous United States using 12 km sized grid cells. Chemical inflow to the 12 km domain was from a global-scale application of the Goddard Earth Observing System-Chemistry model using methodology described in Henderson et al. (2014).

Anthropogenic emissions were based on the 2011 National Emission Inventory (NEI) version 2 (U. S. Environmental Protection Agency, 2016). Emissions in the 2011 NEI are based on a combination of Environmental Protection Agency default information and area-specific information provided by state and local agencies. Biogenic emissions were based on the Biogenic Emission Inventory System version 3.6.1 using the Biogenic Emission Land Use Database version 4.1 vegetation information and hourly Weather Research and Forecasting output temperature and solar radiation as inputs (Bash et al., 2016). Mobile emissions are based on the 2014a version of the Motor Vehicle Emissions Simulator (MOVES2014a; https://www3.epa.gov/otaq/models/moves/). Hour-specific emissions of NO_*x*_ and SO_2_ were used for point sources that reported Continuous Emission Measurement information (largely electric generating units (EGUs)). For all other sources, annual totals from the NEI were converted to hourly model inputs using seasonal, day-of-week, and hour-of-day temporal allocation profiles. The state of Maryland submitted county-specific vehicle fleet information for on-road and nonroad mobile sources and temporal profiles that matched vehicle-type information to typical daily and day of the week travel patterns specific to that area.

CMAQ was run with the Integrated Source Apportionment Method (Kwok et al., 2013, 2015) to estimate contributions of each of 11 specific source sectors to modeled CO, NO_*y*_, and O_3_. Sectors tracked for contribution to primary and secondary pollutants are listed in Table 1. Lateral boundary inflow was also tracked to provide information on the contribution of all sources outside the model domain that include anthropogenic and natural sources in other parts of the United States and beyond.

### 2.3. ΔCO:ΔNO_*y*_ Ratio Derivation

In this analysis we are interested in determining ΔCO:ΔNO_*y*_ ratios where ΔCO and ΔNO_*y*_ signify the increment of these two compounds above regional background within the boundary layer. ΔCO:ΔNO_*y*_ was calculated for modeled and observed values using a linear ordinary least squares regression with CO as the response variable (*y* axis) and NO_*y*_ as the explanatory variable (*x* axis) using the “stats” package in R (R Core Team, 2016). Along with the slope, we also obtained the standard error of each slope, allowing us to calculate a 95% confidence interval, and the *p* value for the significance of the slope (i.e., probability that the slope is different from zero given the standard error). The slope (dCO/dNO_*y*_) is assumed to approximate the enhancement ratio, ΔCO:ΔNO_*y*_, associated with “local” sources (Anderson et al., 2014; Fujita et al., 1992; Mellios et al., 2006; Parrish, 2006). However, without more spatially extensive measurements of CO and NO_*y*_ background mixing ratio, we cannot conclusively determine the geographic extent represented as local emissions.

Three sets of regressions were performed: one set using measured NO_*y*_ and measured CO data, a second set using measured ∑NO_*y*,*i*_ and measured CO data, and a final set using modeled NO_*y*_ and modeled CO outputs. A separate regression was performed for data from each location identified in Figure 1 and each flight day (9 locations × 14 flight days = 126 possible regression data sets). However, 5 day/location combinations did not have data for ∑NO_*y*,*i*_ and 2 days did not have any data collected within the boundary layer over Chesapeake Bay, leaving 124 complete regressions for observed NO_*y*_ and CMAQ NO_*y*_ and 121 complete regressions for ∑NO_*y*,*i*_. Of these regression, there were 121 with statistically significant slopes (*p* value ≤ 0.05) for the observed NO_*y*_, 100 with statistically significant slopes for the observed ∑NO_*y*,*I*_, and 120 with statistically significant slopes for the CMAQ NO_*y*_. In total, 96 site/location pairs had statistically significant slopes for all three regressions. The mean *r*^2^ value for 288 regressions (96 × 3) was 0.70.

In order to emphasize the data most relevant to the surface while not biasing the ΔCO:ΔNO_*y*_ results with contributions from nonlocal sources, we only included data that were collected within the mixed layer as determined using direct meteorological analysis for each flight spiral (Don Lenschow, 2011). For highway flight segments, “onflight” segments (i.e., all measurements taken during P3B flights that were not associated with a spiral) and spirals with no definitive mixed layer determination, the lower of the mixed layers from the previous and subsequent spirals was used. For measurements taken over the Chesapeake Bay, airborne High Spectral Resolution Lidar measurements of aerosol backscatter (accessed at https://www-air.larc.nasa.gov/cgi-bin/ArcView/discover-aq.dc-2011?ANALYSIS=1) were use to determine mixed layer heights. This is in contrast to previous work that assumed a constant 700 m boundary layer during morning hours and 1,500 m during afternoon and evening hours (Anderson et al., 2014). In our analysis, morning mixed layers were mostly above 1,000 m (79% of measurements) and afternoon mixed layers were mostly above 1,500 m (53% of measurements), although there were outliers in both morning and afternoon when the mixed layer was lower than 700 m.

## 3. Results

### 3.1. Characterization of Model Results

#### 3.1.1. Model Performance Evaluation

**Figure 2.**
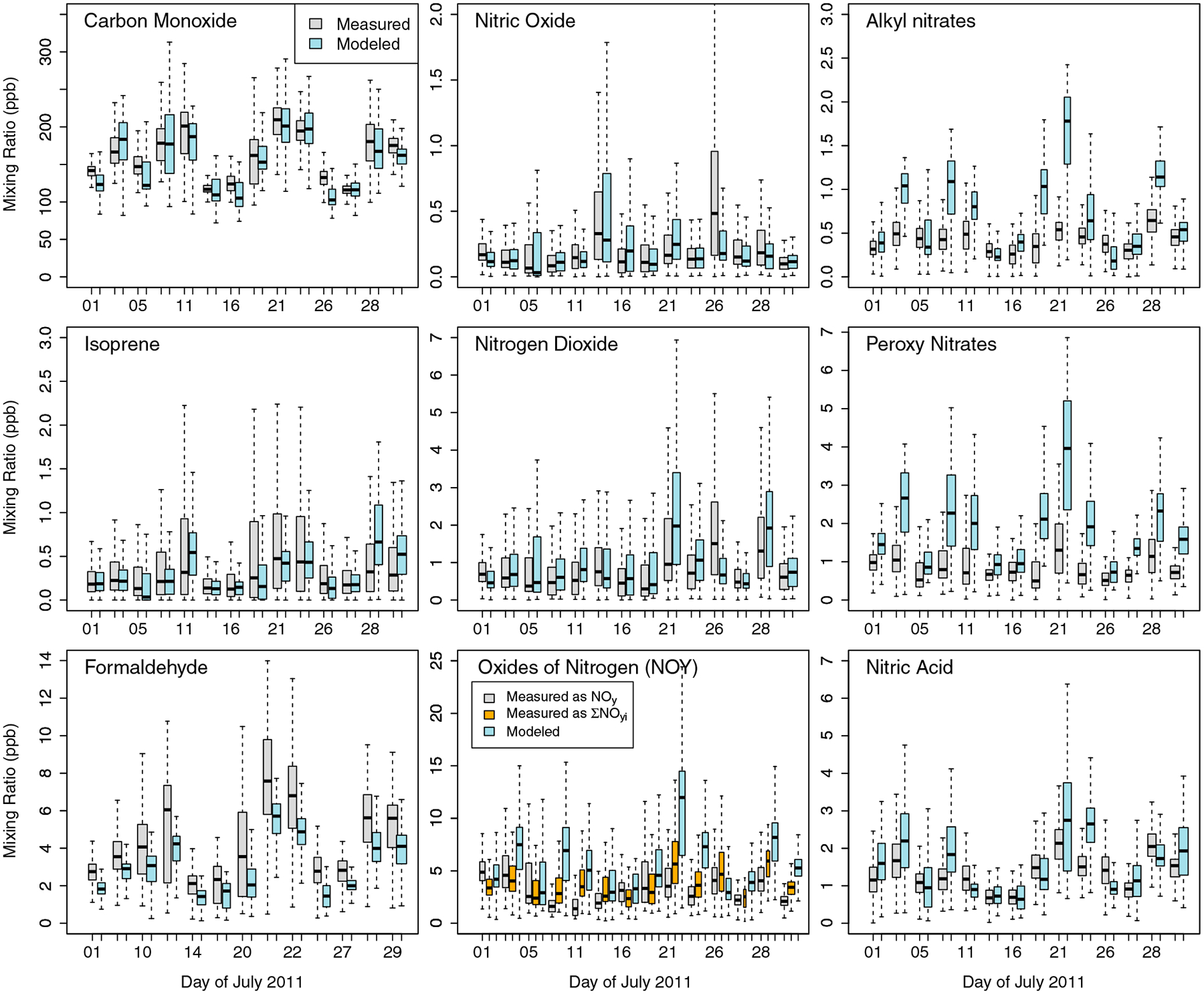
Distribution of measured and hourly modeled carbon monoxide, isoprene, formaldehyde, nitric oxide, nitrogen dioxide, NO_*y*_, alkyl nitrates, peroxy nitrates, and nitric acid by day where the National Aeronautics and Space Administration P-3B was within the mixed layer. The midline shows the median value, boxes indicate the interquartile range, while whiskers extend to 1.5 times the interquartile range. Outliers are not shown.

CMAQ has previously been evaluated in publications including Foley et al. (2010) and Appel et al. (2017). Here we provide brief overview of model performance to verify the model’s value in characterizing air pollution during this episode. First, supporting information Figure S17 shows that the model does a reasonable job at predicting the atmospheric mixed layer at most locations on most days, but it underestimates mixed layer heights over the Chesapeake Bay and across locations on 5, 10, 11, and 20 July. The distributions of daily aircraft measurements are compared with modeled estimates in Figure 2 for isoprene, formaldehyde, CO, and NO_*y*_ species where the aircraft is within the mixing layer. The model does well at predicting the range of measured O_3_ (Figure S14) and CO on most days with normalized mean bias values of 2% and −4%, respectively. Isoprene tends to be underestimated by −23%, suggesting that the isoprene emissions or chemical lifetime are underestimated. Formaldehyde is a major oxidation product of biogenic and anthropogenic VOCs and can be used as a proxy both for biogenic emissions (Curci et al., 2010; Zhu, 2016) and for photochemical activity (e.g., Valin et al., 2016). Formaldehyde model performance is good on some days (e.g., 14, 16, and 28 July) but is biased low on others with a normalized mean bias of −30% across all days. This is not surprising as prior CH_2_O measurement-model studies (e.g., Fried et al., 2011) have shown that unmeasured hydrocarbons, underreported hydrocarbon emissions, and/or additional primary CH_2_O emission sources can all result in model underpredictions for CH_2_O. This is also consistent with findings from Marvin et al. (2017) who found that CH_2_O production with the CB05 chemical mechanism may be biased low by as much as 33% due to insufficient representation of second and late-generation isoprene oxidation. Model performance for several other select organic species is shown in Figure S14. Toluene, an indicator of anthropogenic VOC emissions, is overpredicted on some days and well captured on others.

Due to the intensive measurements available during this field study, we were also able to evaluate speciated and total NO_*y*_ model predictions. NO_2_ mixing ratios are fairly well represented with a mean bias (MB) of 8%. Nitric acid model performance varies from day to day, with good agreement on most days and overpredictions on a few days. Overall, the normalized mean bias for nitric acid is 18%. Figure S14 shows that the model predictions of the ratio of HNO_3_/NO_2_, which can be an indicator used to separate chemistry from emissions, are unbiased in the model except on 10 July when the model overpredicts this ratio, and on 11, 20, and 21 July when the model underpredicts this. Model evaluations for ANs and PNs show that both of these species tend to be substantially overestimated (68% and 118% normalized mean bias, respectively), which may be due to overpredictions of NO_*x*_ emissions, overpredictions of loss rate via reactions with RO_2_, and other uncertainties in the model chemistry. Normalized mean bias across all days for NO_*y*_ is 69% and 50% for ∑NO_*y*,*i*_. It should be noted that NO_*y*_ model performance varies substantially from day to day ranging from moderate underpredictions to extreme overpredictions. As noted in section 3.1.2, we expect that EGU emissions were overestimated during a heat wave on 21 and 22 July. If those 2 days are excluded, the normalized mean bias for NO_*y*_ and ∑NO_*y*,*i*_ drops to 55% and 43%, respectively. Given that model performance for NO_*x*_ and HNO_3_ is reasonable, it appears that most of the NO_*y*_ bias is being driven by aged species such as ANs and PNs. Canty et al. (2015) suggest that the CB05 chemical mechanism used in this modeling may underestimate the photolysis rate of ANs. More recent carbon bond mechanisms that update the AN chemistry have been implemented into CMAQ but were not available with the source apportionment capability at the time of this study. More aggressive destruction of ANs through photolytic reactions would likely decrease modeled AN and improve comparison with ambient measurements although this is unlikely to fully resolve AN overpredictions.

#### 3.1.2. Modeled Source Contributions for CO, NO_*y*_, and O_3_

**Figure 3.**
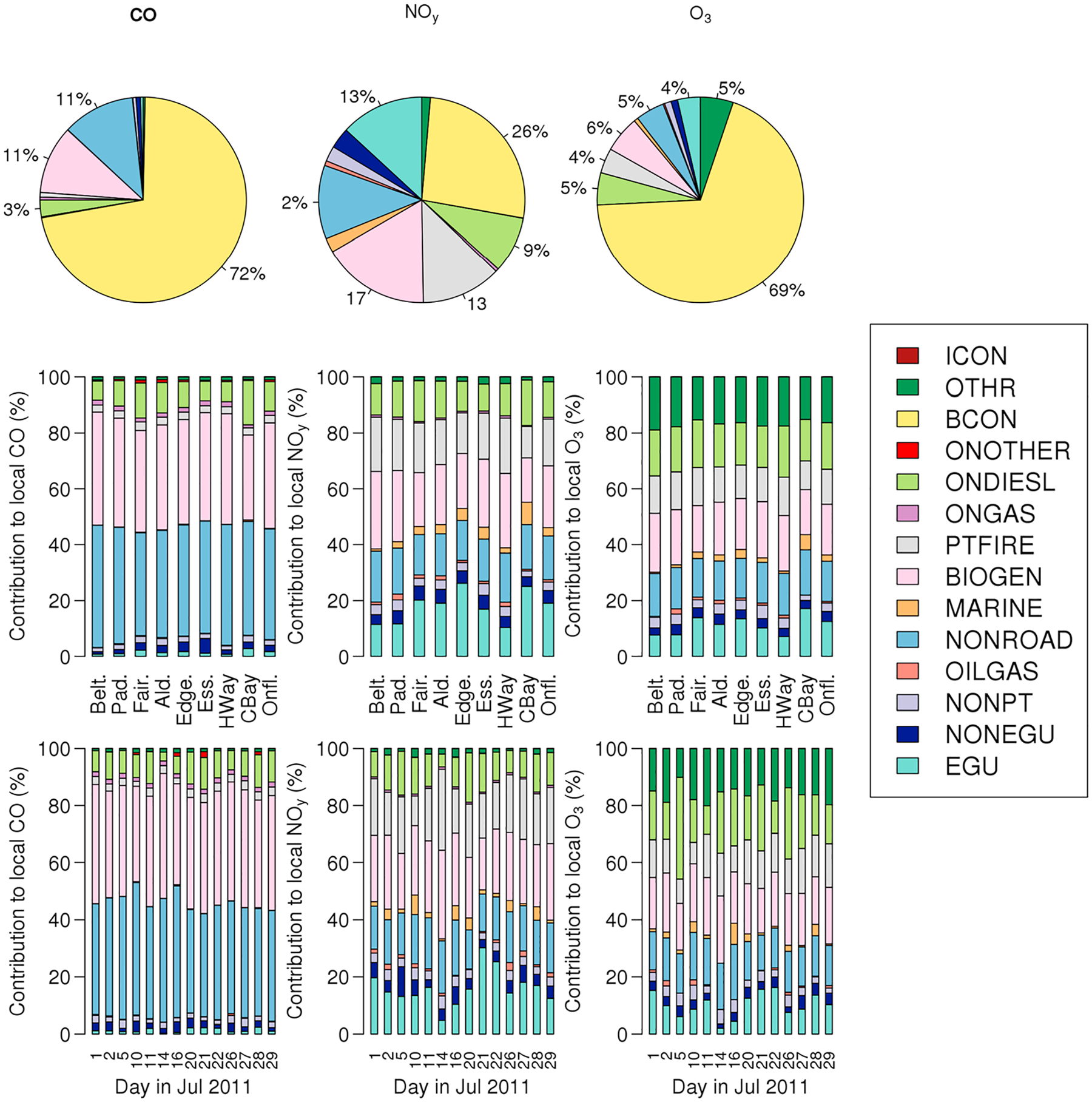
Percent contribution to modeled (left) CO, (middle) NO_*y*_, and (right) O_3_ mixing ratios from different source categories across all locations and days (top row, pie charts). Percent contribution of modeled (left) CO, (middle) NO_*y*_, and (right) O_3_ local mixing ratios (i.e., excluding boundary condition influences) aggregated by location (second row, stacked bar) and by day in July (bottom row, stacked bar).

The source apportionment modeling performed for this analysis provides unique information on modeled source contributions to CO, NO_*y*_, and O_3_ mixing ratios within the mixed layer for the times and locations of aircraft measurements. Figure 3 shows relative contributions from different source categories to mixed layer mixing ratios across all times/locations with P-3B measurements during the DISCOVER-AQ campaign. For the campaign average, integrated across all locations where spirals were flown, boundary conditions (i.e., regional transport) are the largest contributor to all three species. For CO, nonroad equipment (construction equipment, lawn and garden equipment, recreational boats, etc.), on-road gasoline vehicles, and biogenic sources are the next largest source categories accounting for 36%, 34%, and 8% of the local contributions (i.e., contributions excluding boundary conditions). Sources contributing substantially to NO_*y*_ and O_3_ include on-road gasoline vehicles (25% of local contributions for NO_*y*_; 21% of local contributions for O3), on-road diesel vehicles (18% of local contributions for NO_*y*_; 14% of local contributions for O3), nonroad equipment (17% of local contributions for NO_*y*_; 16% of local contributions for O3), EGUs (15% of local contributions for NO_*y*_; 13% of local contributions for O3), and biogenics (13% of local contributions for NO_*y*_; 18% of local contributions for O3). Contributions from certain sources vary by day and location, most notably EGU, non-EGU point source, and marine contributions, but generally the simulated mix of contributing sources is stable in space and time. For instance, emissions from marine vessels contribute most to NO_*y*_ and O_3_ at the Chesapeake Bay, Edgewood, and Essex locations, accounting for 8.0%, 4.3%, and 4.3% of local NO_*y*_ contributions at those three locations and less than 3.5% at all other locations. Additionally, non-EGU point sources have their largest contributions to NO_*y*_ on 5 July (10.3%), the next largest contribution coming on 16 July with only 6.2%. EGU emissions accounted for 10–20% of the total local NO_*y*_ contributions on all days except on 14 July (5%) and 21 and 22 July (30% and 25%, respectively). The larger EGU contributions on 21 and 22 July coincide with a heat wave that resulted in more power plant peaking unit emissions. The emissions spike on 21 and 22 July may be somewhat overestimated, though, as we have found that several municipal solid waste incinerators without Continuous Emission Monitors in the Baltimore area were erroneously temporally allocated like peaking units. While previous work has assumed that the relative contributions of diesel and gasoline sources of on-road NO_*x*_ emissions would be spatially variable, with larger diesel contributions over highway locations and larger gasoline contributions at downwind locations such as Padonia and Aldino (Anderson et al., 2014), the modeling results in Figure 3 do not show any systematic difference in the NO_*y*_ contribution from on-road gasoline versus diesel vehicles by location with 57–60% of the on-road contribution coming from gasoline vehicles at all locations except for Fairhill where gasoline vehicles only accounted for 52% of the on-road contribution. The modeled surface level wildland fire impacts are minimal.

### 3.2. Measured and Modeled ΔCO:ΔNO_*y*_ Ratios

Table S2 in the supporting information provides ΔCO:ΔNO_*y*_ values from all regressions using measured NO_*y*_ and CO, measured ∑NO_*y*,*i*_ and CO, and modeled NO_*y*_ and CO for each location and flight day combination. ΔCO:ΔNO_*y*_ geometric mean values (± mean of the standard errors in all regression) across all regressions with significant slopes were 9.8 ± 1.0, 8.3 ± 1.3, and 9.5 ± 0.5 for measured NO_*y*_ and CO, measured ∑NO_*y*,*i*_ and CO, and modeled NO_*y*_ and CO, respectively. Our analysis of observed ΔCO:ΔNO_*y*_ has geometric mean values similar to previous analysis (Anderson et al., 2014; ΔCO:ΔNO_*y*_ = 11.2 ± 1.2).

While the use of measured NO_*y*_ versus ∑NO_*y*,*i*_ did not substantially impact the derived ratios in all cases, in some cases the slopes for regressions using these two measured NO_*y*_ values differed by up to a factor of 3.4. Approximately one third of the significant regression slopes (31 out of 96 regressions) had slopes that were significantly different (i.e., 95th percentile confidence intervals of the slopes did not overlap) when using NO_*y*_ versus ∑NO_*y*,*i*_ (see Table S2) suggesting substantially different inferred emission ratios. Consequently, these 31 pairs are excluded from further analysis. Even within the remaining regressions, the slopes using the two different measurement methods vary on average by 22% and by up to 90%. Any comparison of observed, modeled, or emitted CO:NO_*y*_ should be made keeping this uncertainty in mind.

**Figure 4.**
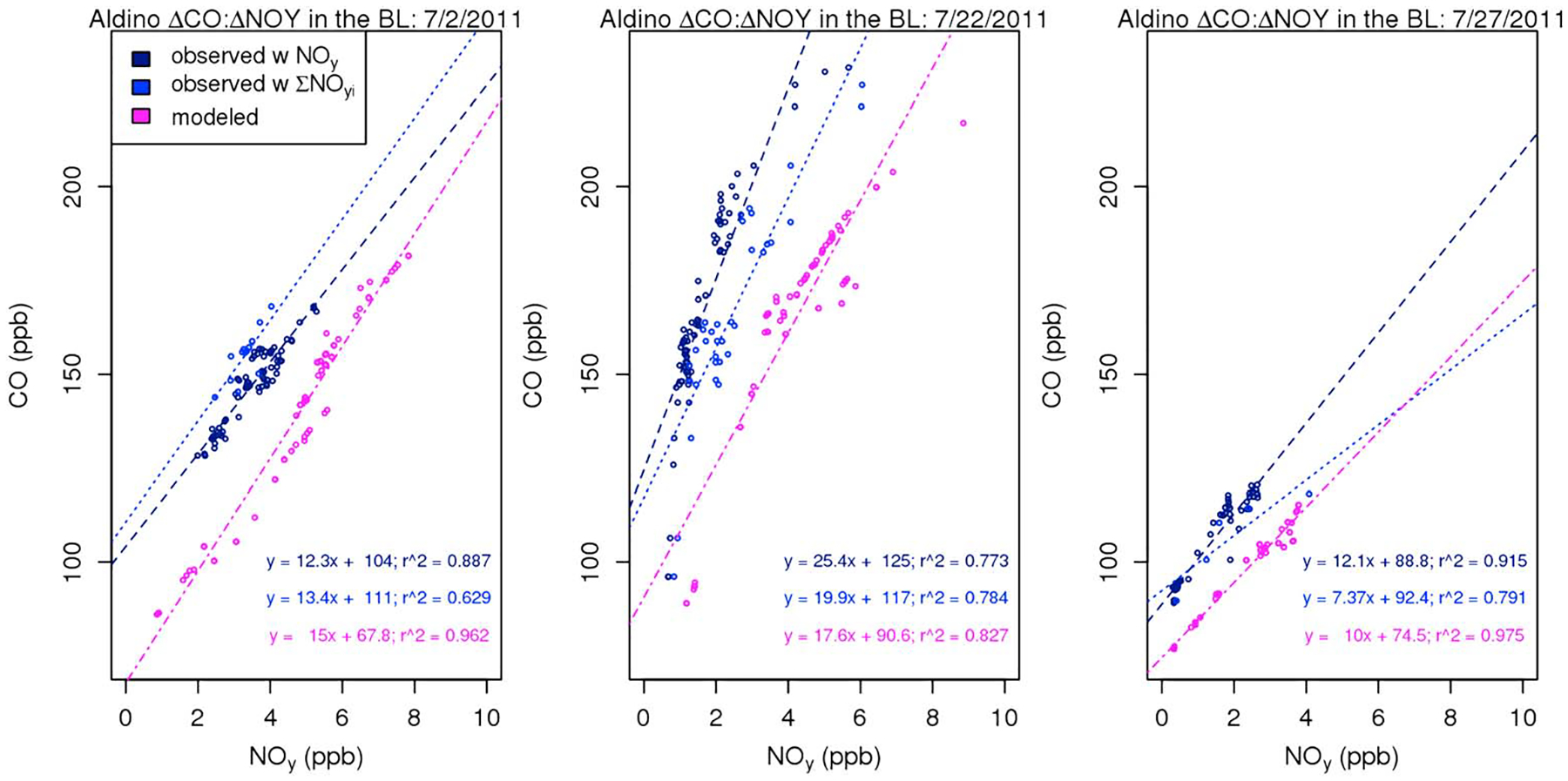
CO and NO_*y*_ values as associated regression lines for data over Aldino on (a) 2 July 2011, (b) 22 July 2011, and (c) 27 July 2011.

The modeled and measured ΔCO:ΔNO_*y*_ values agree quite well on some days and locations while they are very different for others as demonstrated in Figure 4 that shows regressions for the measured ΔCO:ΔNO_*y*_, measured ΔCO:Δ∑NO_*y*,*i*_, and modeled ΔCO:ΔNO_*y*_ on three different days at Aldino. For Aldino, the largest model underpredictions generally occur on days with the highest observed ΔCO:ΔNO_*y*_ ratios (days with observed ΔCO:ΔNO_*y*_ > = 15). On many of those days, using the ∑NO_*y*,*i*_ instead of the NO_*y*_ measurement reduced the discrepancy (see Figure 4, middle) although it did not close the gap entirely.

Note that while a previous study reported that they were unable to perform regressions on modeled data due to essentially constant CO and NO_*y*_ concentrations within the boundary layer (Anderson et al., 2014), this issue was not a problem in our model output. As demonstrated by Figure 4, the model shows a great deal of variability in both CO and NO_*y*_ concentrations within the boundary layer when matched to spiral times and locations on each day at Aldino. Figures S21–S23 in the supporting information provide time series for the full flights on 2, 22, and 27 July of both modeled and observed CO and NO_*y*_ to demonstrate that the results in Figure 3 are not unique to Aldino. Those figures show that the model did capture CO and NO_*y*_ variability throughout the aircraft track when samples were taken within the boundary layer.

**Figure 5.**
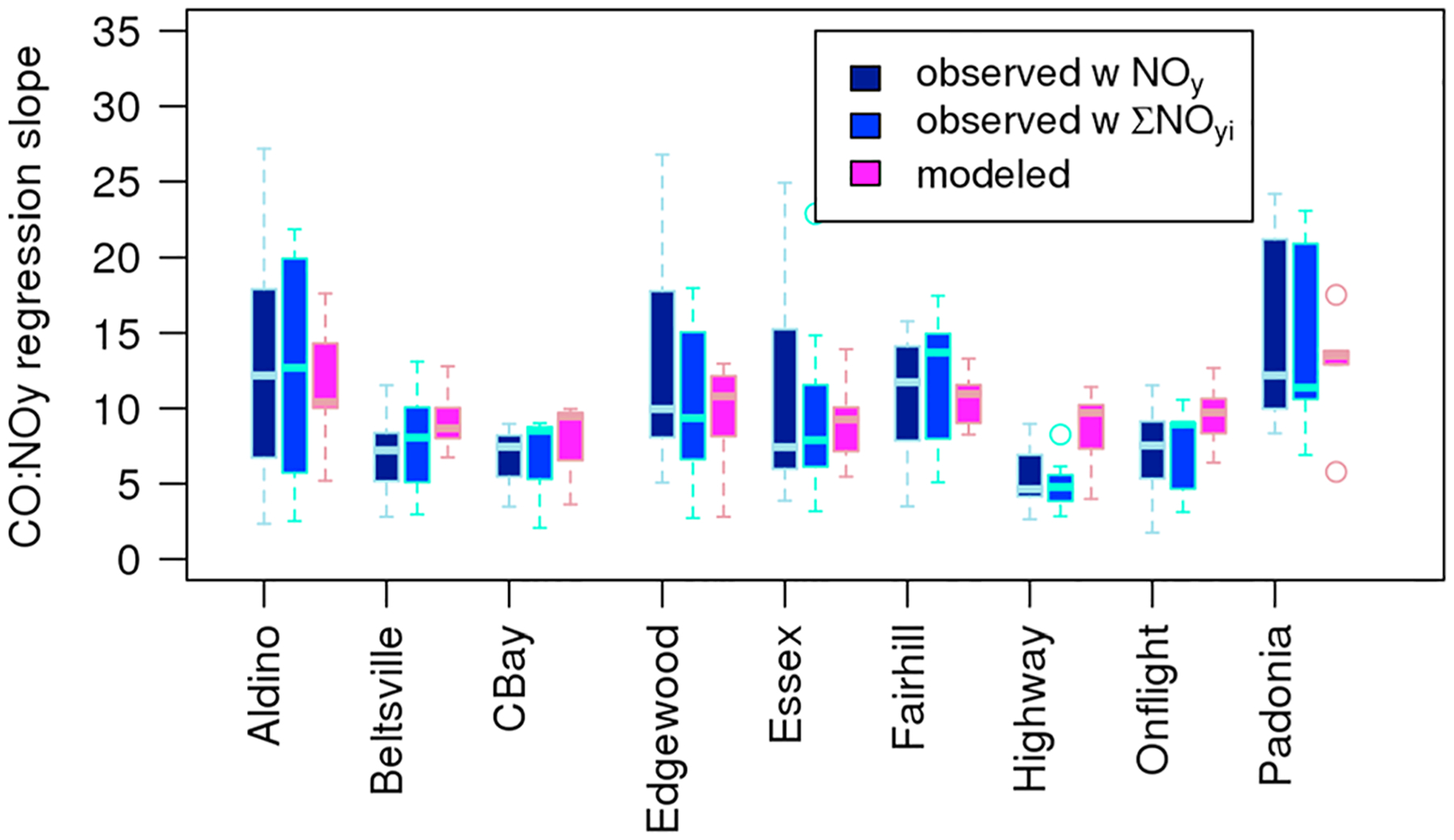
Boxplot showing range of daily ΔCO:ΔNO_*y*_ regressions at each site for the subset of 65 regressions that had statistically significant slopes for all three data sets and for which the 95th percentile confidence interval for the NO_*y*_ and the ∑NO_*y*,*I*_ regression slopes overlapped. The midline shows the median value, boxes indicate the interquartile range, while whiskers extend to 1.5 times the interquartile range. Circles indicate outlier values.

Figure 5 compares the distribution of ΔCO:ΔNO_*y*_ values using measured NO_*y*_, measured ∑NO_*y*,*i*_, and modeled mixing ratios at each location. Figure 5 includes the 65 location-day pairs for which all three regressions had statistically significant slopes and for which the slopes for the NO_*y*_ and ∑NO_*y*,*i*_ were within each other’s 95th percentile confidence interval (i.e., excluding 31 regressions where the two measurement methods were statistically different as shown by orange boxes in Table S2). There were between 6 and 10 days included in the boxplots for all sites except for Chesapeake Bay that only had 3 days that met these criteria. Geometric mean measured ΔCO:ΔNO_*y*_ values at each location for both measurement methods range from 4.9 to 13.6 for measured NO_*y*_, 4.7 to 12.9 for measured ∑NO_*y*,*i*_, and 7 to 12.2 for modeled data. Highway locations have the lowest geometric mean measured ΔCO:ΔNO_*y*_ (NO_*y*_: 4.7; ∑NO_*y*,*i*_: 4.9). The spiral location closest to the source region of Washington D.C. (Beltsville) along with the onflight and Chesapeake Bay measurements has the next lowest geometric mean measured ΔCO:ΔNO_*y*_ values (NO_*y*_: 6.1–6.3; ∑NO_*y*,*i*_: 5.4–7.0). The downwind and suburban locations (Edgewood, Fairhill, Aldino, and Padonia) all have substantially higher measured geometric mean ΔCO:ΔNO_*y*_ values between 9.0 and 13.6. While there are spatial differences in CO and NO_*y*_ source contributions (section 3.1.2), there does not appear to be consistent differences in emission source types contributing to the three source regions with the lowest ΔCO:ΔNO_*y*_ values versus the four downwind locations with higher ΔCO:ΔNO_*y*_ values, suggesting that atmospheric processing may be increasing ΔCO:ΔNO_*y*_ as the air mass ages.

We performed Welch’s *t* tests (Welch, 1947) on the distribution of measured and modeled slopes at each location to determine whether they were statistically different. We found that the highway was the only location where the difference in measured and modeled slopes was statistically significant for *p* < 0.05 (geometric mean of 4.9 for regressions using measured NO_*y*_, 4.7 for regressions using measured ∑NO_*y*,*i*_, and 8.3 for regressions using modeled data). The null hypothesis that the true difference in means is not equal to 0 could not be rejected for any of the spiral locations or for the onflight and Chesapeake Bay measurements. These results were consistent across both NO_*y*_ and ∑NO_*y*,*i*_. So while the model does appear to replicate the larger ΔCO:ΔNO_*y*_ values at the downwind spiral locations, it only showed slightly lower values over the highway while the measurements indicated much lower values over the highway. It is possible that even at a 4 km grid resolution and hourly time integration, the model does not fully capture the extremely fresh emissions being measured by the aircraft over the highway and may be mixing and aging those emissions compared to measurements made close to the emissions source.

While site-to-site variability in measured and modeled slopes were similar, the day-to-day variability in modeled ΔCO:ΔNO_*y*_ values were much less than for measured ΔCO:ΔNO_*y*_. The interquartile range of modeled ΔCO:ΔNO_*y*_ at each of the aircraft spiral locations shown in Figure 5 was far smaller than the interquartile range for measured values. Since the sources are not expected to change substantially from day to day (see Figure 3), this may indicate that there is some meteorology-driven variability driving this ratio that the model is not completely replicating. For example, it is possible that the model cannot capture extreme ΔCO:ΔNO_*y*_ values due either to its temporal and spatial resolution compared to 1 s measurements at a point in space or the model diluting emissions too rapidly.

### 3.3. Drivers of Variability in ΔCO:ΔNO_*y*_

In the last section, we noted much more variability in the measured ΔCO:ΔNO_*y*_ than in the modeled ΔCO:ΔNO_*y*_. In this section we explore the various processes that can impact this ratio in the mixed layer: (1) emissions, (2) vertically and temporally varying background concentrations of CO and NO_*y*_, and (3) photochemical aging.

#### 3.3.1. Emissions Sources

Emitted ratios from different source categories vary by 3 orders of magnitude (Table 1) and span the measured ΔCO:ΔNO_*y*_ values. Information from this table can be combined with the source contributions shown in Figure 3 to estimate what the modeled ΔCO:ΔNO_*y*_ would be due solely to contributions of varying NO_*x*_ and CO emissions from different sources. Here we use the emitted CO:NO_*x*_ based on aggregated emissions from the seven counties sampled in the field campaign shown in Table 1, which ignores some spatial and temporal variability in this ratio. We then calculate what the modeled ΔCO:ΔNO_*y*_ would be based on a linear combination of emission sources (i.e., the overall emitted ratio at location, *l*, and day, *d*: *r*_TOT,*ld*_ for a given day and location), assuming that emitted molar fractions from each source impacting that day and location are equivalent to modeled mole fraction from Figure 3 for that day and location. We use equation (1), where *X*_CO,*i,ld*_ and *X*_NO*y,i,ld*_ represent the modeled mole fractions of CO and NO_*y*_ from source category *i* at location, *l*, and day, *d*. The emitted molar ratio of CO:NO_*x*_ from source category *i* is *r*_*i*_. Note that the total emitted ratio impacting location *l* on day *d*, *r*_TOT,*ld*_, can be calculated either using the modeled mole fractions of CO or NO_*y*_ that lead to identical answers if emitted CO and NO_*y*_ are only impacted by mixing and not being enhanced or reduced by other atmospheric processing.


(1) $$r_{\text{TOT},ld}=\frac{1}{\sum_{i}\left(\frac{X_{\text{CO},i,ld}}{r_{i}}\right)}=\sum_{i}\left(X_{\text{NO}y,i,ld}\times r_{i}\right)$$

**Figure 6.**
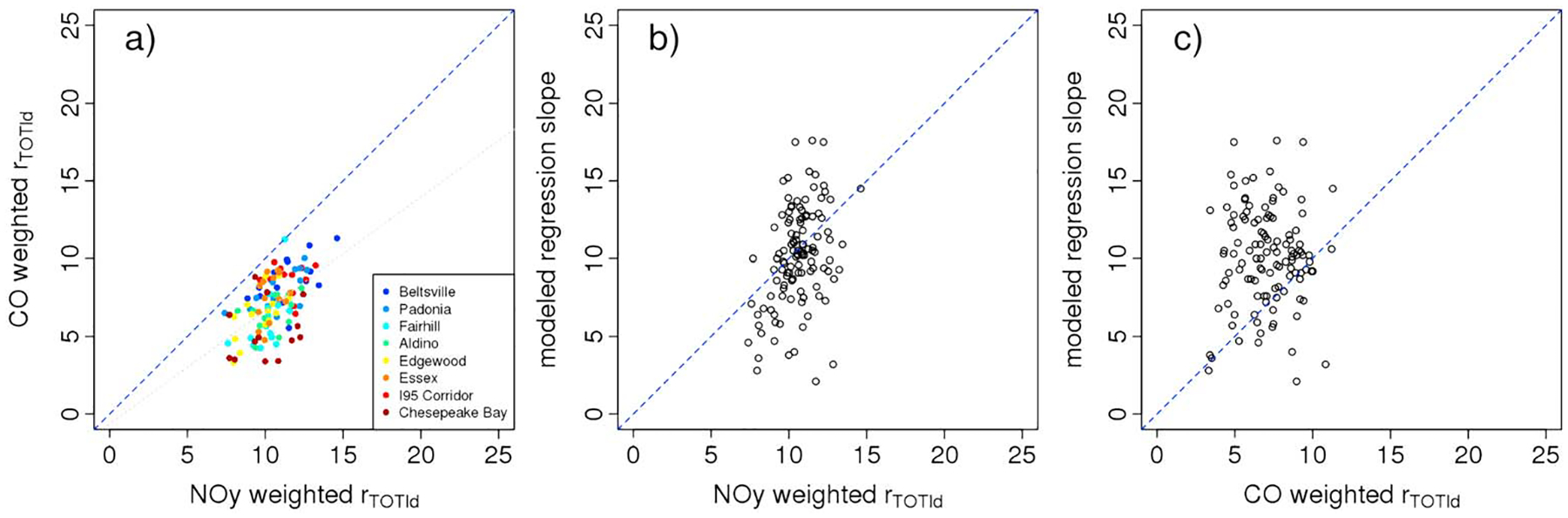
(a) Comparison of *r*_TOT,*ld*_ calculated using NO_*y*_ versus CO mole fractions, (b) comparison of *r*_TOT,*ld*_ calculated using NO_*y*_ mole fraction to modeled regression slopes, and (c) comparison of *r*_TOT_ calculated using CO mole fraction to modeled regression slopes. Blue dashed lines indicate 1:1 line. Gray dashed line in Figure 6a shows regression line of best fit.

**Figure 7.**
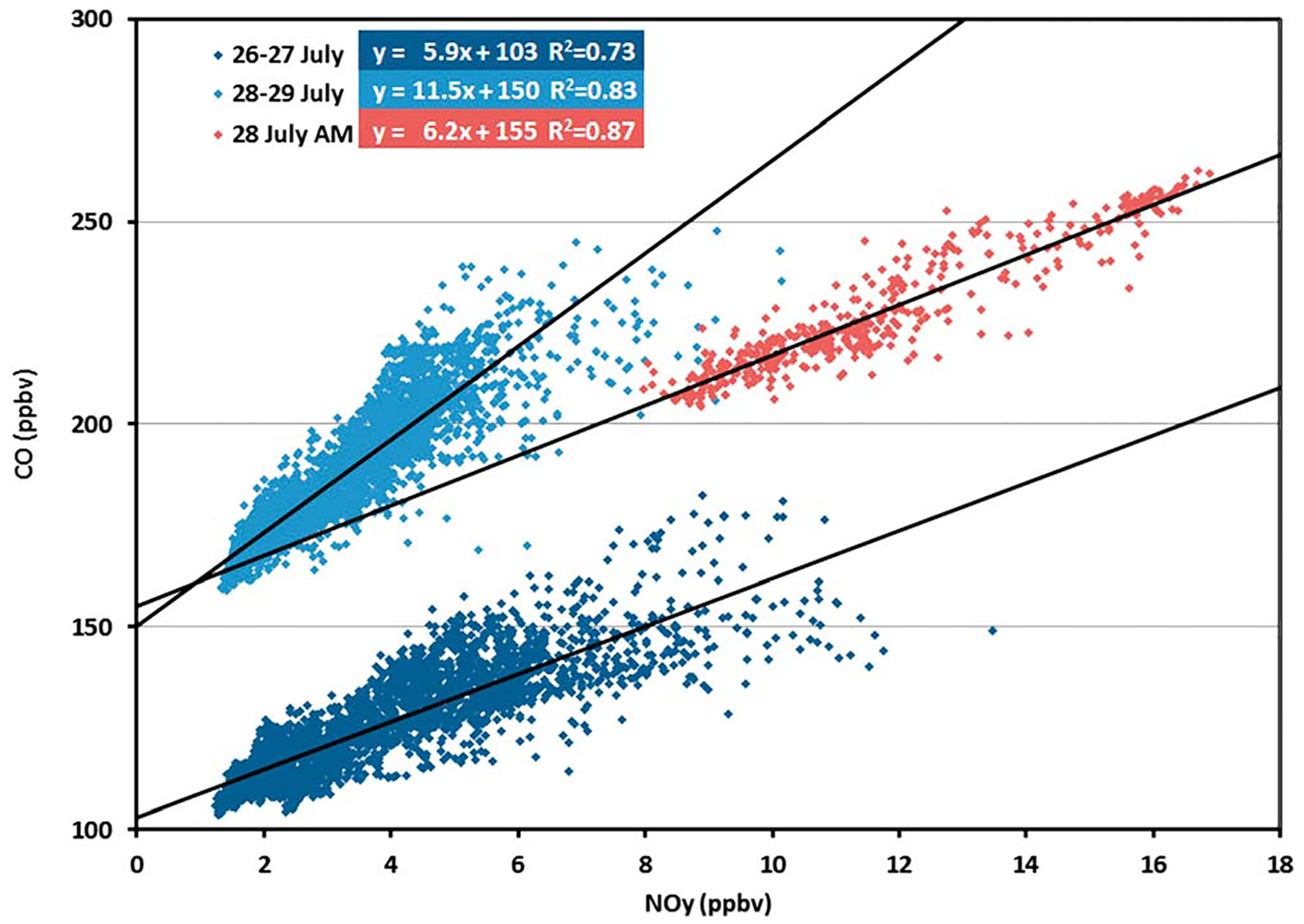
CO:NO_*y*_ regressions from P3 overpasses of the highway for periods of lower biogenic activity (July 26–27 and July 28 a.m.) and higher biogenic activity (28 July p.m. to 29 July).

Figure 6a shows the comparison of *r*_TOT,*ld*_ calculated using the modeled NO_*y*_ mole fraction compared to the modeled CO mole fraction. Each data point represents a single day/location pair. There is a systematic difference in *r*_TOT,*ld*_ calculated with these two methods demonstrating that CO and NO_*y*_ tagged concentrations are impacted by processes other than emissions and mixing such that atmospheric ΔCO:ΔNO_*y*_ values alone cannot be used directly to validate emission profiles. As might be expected, data points from locations closest to emission sources (I-95 corridor and Beltsville) generally fall closest to the 1:1 line, while data points derived from downwind locations for which the air mass has had more time to age (Chesapeake Bay, Fairhill, Aldino, and Edgewood) tend to fall further from the 1:1 line. Figures 7b and 7c compare *r*_TOT,*ld*_ to the modeled regression slopes. When the *r*_TOT,*ld*_ is calculated using NO_*y*_ mole fraction, the correlation with the regression slopes is 0.38 (*r*^2^ = 0.14) suggesting that only 14% of the variation in modeled regression slopes can be explained by variation in emitted ratios and source strengths. In contrast, when *r*_TOT,*ld*_ is calculated using CO mole fraction, the correlation is −0.03. This implies a stronger relationship between tagged NO_*y*_ concentrations and emitted source strengths than tagged CO concentrations and emitted source strengths suggesting that CO is disproportionately impacted by secondary formation and potentially regional to continental-scale inflow.

We further explore the relationship between emitted and modeled concentrations by looking at the ratio (regression line slope, see Figure S24) of tagged CO to tagged NO_*y*_ in the model for four major source categories at each spiral location and comparing emitted ratios (Table 2). If atmospheric processing is not significant, tagged contribution ratios should be equal to emitted ratios. Table 2 shows that sector-tagged modeled ratios for nonroad, on-road gasoline, and on-road diesel sectors are substantially higher than the emitted ratios. Using the average seven-county emissions totals, ambient nonroad, on-road gasoline, and on-road diesel emissions are enhanced in the model atmosphere compared to their emission values by 5–37%, 9–28%, and 20–50% respectively. There are two possible explanations for this difference: (1) spatial variability in sources could lead to the seven-county average not being representative of the impact of a local source on a specific location and (2) the ΔCO:ΔNO_*y*_ ratio is not conserved from the emission point to the modeled value in the atmosphere due to atmospheric processes (i.e., secondary CO formation or preferential NO_*y*_ deposition) discussed in section 3.3.2. The first hypothesis is plausible for the nonroad category due to the large variability in CO:NO_*y*_ across different emission sources within this category. For instance, the nonroad tag may be more heavily weighted toward diesel sources in areas with a lot of construction activity and more heavily weighted toward gasoline sources in residential areas with a large amount of emissions from lawn and garden activity. However, for both the on-road gasoline and on-road diesel categories, there is little variability in the emitted CO:NO_*y*_ ratio. This suggests that atmospheric processing in the model enhances the ratios above emitted values precluding the use of ambient ratios to infer direct relationships with emission ratios. There are several possible explanations why EGU modeled ratios do not appear to be enhanced above emitted ratios. First, similar to nonroad emissions, there is a large degree of spatial variability in emitted CO:NO_*x*_ from different EGU source types. Second, unlike mobile emissions, EGU emissions occur aloft so there is less opportunity for NO_*y*_ deposition to the surface. Third, EGU emissions generally occur in less urbanized areas than mobile source and therefore may not be exposed to high concentrations of atmospheric oxidants, leading to less aging. Finally, also unlike mobile emissions, EGU sources include little to no VOC emissions that can react to form secondary CO. This suggests EGU emissions are less likely to be impacted by atmospheric processing and explains why modeled ratios from tagged EGU CO and NO_*y*_ are in the same range as emitted EGU ratios. If in fact the use of CB05 chemistry results in an underestimate of the formation of CH_2_O from VOCs and ultimately CO, as reported by Marvin et al. (2017), the atmospheric enhancements of ΔCO:ΔNO_*y*_ over emitted ratios could be even larger. The main Table 2 numbers for CO: NO_*y*_ ratios in the CMAQ source apportionment modeling are derived from ordinary least squares regressions consistent with the methods described in section 2.3. To test the dependence of these results on the choice of regression methodology, these calculations were redone using orthogonal regression that accounts for measurement uncertainty in both the dependent and independent variables (shown in parentheses). Results derived from the orthogonal regressions show even larger enhancements above the emitted values and confirm that the results are not an artifact of the chosen regression methodology.

#### 3.3.2. Sampling Distinct Air Masses: Boundary Layer Entrainment of Free Troposphere

Vertical variations of background CO and NO_*y*_ can mask the covariations (i.e., ΔCO:ΔNO_*y*_) driven by local emissions. While the slope of the regression would not be impacted by background pollutant concentrations so long as CO and NO_*y*_ background mixing ratios are vertically uniform, the regression is not able to account for variations driven by the independently varying background CO and NO_*y*_ mixing ratios. To further demonstrate the impact of sampling boundary layer and free tropospheric air on regression results, we performed sensitivity analysis by filtering data included in the regressions using a constant altitude cutoff (500 m, 1,000 m, 2,000 m, or 3,000 m), rather than a varying boundary layer height used in the previous sections. This sensitivity analysis found that as the altitude cutoff increased (i.e., data from higher altitudes were included in the regressions), ΔCO:ΔNO_*y*_ systematically increased. This finding highlights the importance of restricting the regressions to only data that were collected within the boundary layer, since regressions that included data points taken from higher altitudes increasingly incorporate free tropospheric air where variations in background CO can be large relative to the low NO_*y*_ values, which can skew the results. To address this limitation, in the analyses presented in sections 3.1, 3.2, and 3.3.1 we selected only data collected within the mixed layer. However, the large contributions of boundary inflow to CO and NO_*y*_ even within the mixed layer shown in Figure 3 highlight the potential for variability in the regional background to impact the regressions allowing for the possibility that selecting for data collected within the mixed layer may not completely eliminate the impact of vertical entrainment on ΔCO:ΔNO_*y*_ ratios.

Background sources are the primary contributor of the total summertime CO over the eastern United States (e.g., Hudman et al., 2008 and Figure 3 of this work), and CO is long-lived (τ_CO + OH_ ~ 30 days). In contrast, the lifetime of atmospheric NO_*y*_ to dry and wet deposition is much shorter (τ_NO*y* dep_ < 2 days), although Figure 3 demonstrates that regionally transported NO_*y*_ still constitutes a substantial portion of NO_*y*_ in the mixed layer. As a result, regionally processed air masses in the summertime east United States are enriched in CO relative to NO_*y*_. As discussed above, analyses of local CO:NO_*y*_ emission ratios using measurements of ambient mixing ratios should be unaffected by these regional variations so long as they are negligible relative to total ΔCO and ΔNO_*y*_. This in reality, however, is very challenging. Measurements sample distinct layers such as the local boundary layer, residual layers, and the free troposphere, which reflect both local process and the historical differences between the distinct layers.

Furthermore, there are strong vertical gradients of horizontal wind speeds, especially at nighttime, that when followed by morning time boundary layer growth incorporate locally impacted air masses with residual or free tropospheric air. To determine if these effects are observable in the daytime DISCOVER-AQ data, we looked at the correlation coefficient of all 1 s NO_*y*_ and CO measurements below 1 km altitude and between 12 p.m. and 1 p.m. (note that this is not the same selection criteria used for the regression shown in section 3.2) and found that the *R*^2^ for this subset is only 0.25. However, the correlation increased when we looked at individual spirals within this data subset (*R*^2^ = 0.54 ± 0.28; *μ* ± *σ*). The increase in correlation in individual spirals builds confidence that selecting data from isolated days and locations can reduce the influence of variations driven by larger-scale atmospheric processes and improve our ability to isolate a signal from local emissions. However, within these individual spirals we also find that the correlation of CO with H_2_O in individual profiles is strong (*R*^2^ = 0.49 ± 0.31; *μ* ± *σ*), suggesting that there is some influence of dynamical processes on the CO mixing ratios. These findings indicate that within the lowest 1 km of the atmosphere at midday, the influence of variations driven by larger-scale atmospheric processes may still impact CO mixing ratios and therefore the derived ΔCO:ΔNO_*y*_.

#### 3.3.3. Chemistry

Atmospheric processing through chemistry and deposition impacts CO and NO_*y*_ differently. CO has a much longer atmospheric lifetime than NO_*y*_ with respect to deposition, so air masses near the ground become enriched in CO relative to NO_*y*_ as they age. In addition, secondary sources of CO from biogenic and anthropogenic VOC oxidation also enhance CO relative to NO_*y*_ as the air mass ages. Both of these processes result in larger ΔCO:ΔNO_*y*_ ratios as air masses age.

The period of 26–29 July provides an excellent example of ΔCO:ΔNO_*y*_ variability that is driven by chemistry rather than emissions. Data for ΔCO:ΔNO_*y*_ during this period is shown in Figure 7. These data are high-resolution (1 Hz) observations taken from the low-altitude (305 m) transects along the interstate highway (I-95 and I-695) between Beltsville and Padonia. These data from the highway sampling are used for two reasons: (1) the data always fall in the boundary layer and (2) the 1 Hz data exhibit high-frequency fluctuations in CO and NO_*y*_ that cannot be examined with the coarser resolution of the ∑NO_*y*,*i*_ data or the model.

ΔCO:ΔNO_*y*_ for this period falls into three distinct populations exhibiting shifts in the relationship between 26 and 27 July, early morning on 28 July, and remaining observations for 28 and 29 July. These changes can be described in terms of the regressions of CO to NO_*y*_ that show changes in both ΔCO:ΔNO_*y*_ and the background conditions for CO. There appear to be two distinct shifts in the data. First, between 26 and 27 July and the morning of 28 July, there appears to be a shift in background CO demonstrated by the regression line shifting upward by 50 ppb without a corresponding change in the slope. One important aspect of the observations on the morning of 28 July is the average NO_*x*_/NO_*y*_ ratio of 0.91, which is substantially higher than the average value of 0.57 observed for the rest of the data for this 4 day period. Average NO_*y*_ is also observed at mixing ratios much greater than at other times (11.7 ppbv versus 3.6 ppbv). This suggests minimal chemical processing for these emissions compared to observations at other times. These two phenomena are consistent with meteorological conditions occurring on 27 and 28 July. On 27 July, a cold front approached Baltimore from the north bringing air with relatively lower background CO concentrations. Between midnight and 10 a.m. on 28 July, the front moved back northward bringing air from the southeast, a region with high biogenic emissions and therefore higher background CO concentrations. After this frontal passage, winds were calm allowing fresh NO_*x*_ to build up from local sources. Second, a distinct shift in ΔCO:ΔNO_*y*_ occurs between the morning of 28 July and the afternoon of 28 July with the ΔCO:ΔNO_*y*_ slope doubling from 5.9 to 11.5 accompanied by lower concentrations of both CO and NO_*y*_. The lower CO and NO_*y*_ concentrations are likely due to increased dilution with a growing afternoon boundary layer. There is no observed change of background CO between the morning and the afternoon of 28 July. A possible explanation for this change in slope is related to additional chemical production of CO from increased biogenic VOC emissions. This secondary production of CO from biogenic VOCs occurs rapidly (on the time scale of 1–3 h) in the presence of NO_*x*_ (e.g., Schroeder et al., 2016; Valin et al., 2016; Wolfe et al., 2016). As observed by the aircraft at 305 m, average temperatures steadily rose from 296.5 to 305.4 K from 26 to 29 July. This was accompanied by an increase in observed isoprene by more than a factor of 2 (290 to 675 pptv) and increases in CH_2_O (the key intermediate species between isoprene and CO) from 2.8 ppbv to 6.6 ppbv from 26–27 July to 28–29 July. Calculations using the box model described in Schroeder et al. (2016) indicate that secondary production of CO doubles from 13 to 26 ppbv per day.

**Figure 8.**
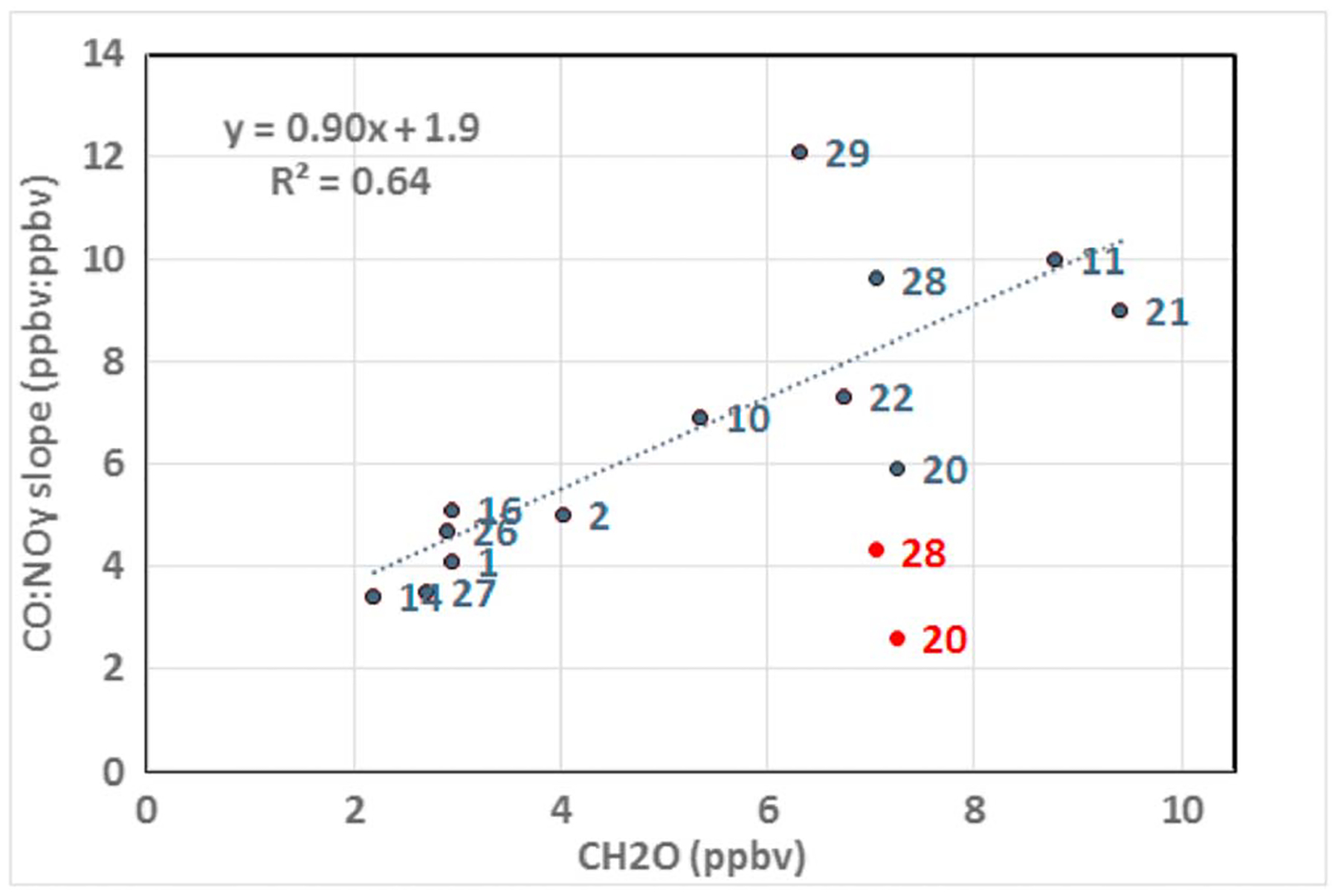
CO:NO_*y*_ slopes from Table S2 for highway NO_*y*_ data versus average CH_2_O. Data points are labeled by the date in July. Red data points for 20 and 28 July indicated original regressions from Table S2 that include distinct populations of high NO_*y*_ data (e.g., see a.m. data for 28 July in Figure 7). Blue points for those data indicate regressions with high NO_*y*_ data removed. The regression line does not include the red points.

To further investigate the possibility that secondary CO from biogenic VOC oxidation may impact ΔCO:ΔNO_*y*_, we look at the relationship between observed ΔCO:ΔNO_*y*_ highway values from Table S2 and average CH_2_O observed during highway sampling on each day. Figure 8 shows the strong relationship between the ΔCO:ΔNO_*y*_ and CH_2_O. This relationship improves when removing data points from 20 and 28 July, both of which exhibited significant changes between morning and afternoon periods due to large values of NO_*y*_ not typically observed. These 2 days account for 90% of NO_*y*_ observations greater than 12 ppbv. ΔCO:ΔNO_*y*_ slopes including these high NO_*y*_ populations are shown in red. These observations are consistent with results from Buhr et al., 1992 who found that ΔCO:ΔNO_*y*_ increased with NO_z_ mixing ratios indicative of aged air masses and suggested that atmospheric processing leads to increase in ΔCO:ΔNO_*y*_.

## 4. Discussion

Understanding the limitations in interpreting the ΔCO:ΔNO_*y*_ ratio as derived from ambient aircraft data is important in the larger context of the community’s recent efforts to evaluate NO_*x*_ emissions from various source types. Similar to previous work by Anderson et al. (2014), Canty et al. (2015), and Travis et al. (2016), the modeling presented in this paper found an overestimate of modeled NO_*y*_ when compared to ambient estimates. However, determining the source of model errors is not straightforward as the model and ambient concentrations are impacted by emissions, chemistry, deposition, mixing, and transport. The large day-to-day variability in model NO_*x*_ performance shown in Figure 2 (from −22% to +285% normalized mean bias on different days using the NO_*y*_ measurements and from −43% to +105% normalized mean bias on different days using the ∑NO_*y*,*i*_ measurement), combined with the fact that emissions do not vary substantially from day to day, suggests that meteorology is a confounding factor in the NO_*x*_ overpredictions.

Several studies have tried to use comparisons between observations and model estimates to constrain on-road NO_*x*_ emissions. Multiple studies (Anderson et al., 2014; Canty et al., 2015) have used CMAQ model simulations of the Baltimore area in July 2011 to conclude that mobile source NO_*x*_ emissions in the 2011 Environmental Protection Agency modeling platform were too high by 50–70%. Researchers using satellite data to constrain emission estimates through model sensitivity analysis concluded that mobile source NO_*x*_ emissions over Texas in the summer of 2013 were overpredicted by approximately 30% (Souri et al., 2016). Travis et al. (2016) found that they needed to reduce all non-EGU emissions by 60% in order to have modeled NO_2_ columns match satellite retrievals during the summer of 2013 in the southeast United States. However, Travis et al. (2016) also found that even with reduced emissions, the vertical profile of NO_*y*_ component species, which is impacted by mixing and chemistry, did not match vertical profiles derived from aircraft measurements. Interpretation of these results is complicated by the fact that the column totals retrieved by satellites are highly sensitive to assumptions about vertical NO_2._ The range in the emission evaluation results from these different studies highlights the difficulty in quantifying the bias with a single summary statistic in light of the complex interactions between physical and chemical processes in the atmosphere and in the model.

A recent analysis compared modeled NO_*x*_ concentrations against ambient measurements in multiple seasons and found that, on average in the summer, modeled NO_*x*_ concentrations were unbiased during the daytime across the United States but were high overnight and during morning and evening transition periods (Appel et al., 2017). Uncertainties in model treatment of boundary layer mixing during these times makes it difficult to ascertain if these model NO_*x*_ overpredictions were the result of emissions, mixing, or both. Further, Appel et al. (2017) found that the nighttime NO_*x*_ bias was largely gone or even reversed (a model underestimate) at other times of year. This seasonal dependence of model NO_*x*_ performance suggests meteorological factors or summertime chemistry may be playing an important role in any discrepancies between modeled nitrogen species and observations. This suggests studies focused on emission inventory validation using ΔCO:ΔNO_*y*_ ratios should seek complimentary data that are less impacted by photochemistry (e.g., cold season measurements). While the work by Anderson et al. (2014) who compared observed ΔCO:ΔNO_*y*_ ratios directly to emissions was intended avoid confounding impacts of meteorology and chemistry, we find that this metric is impacted by those processes making interpretation of those comparisons problematic.

While the use of photochemical models to constrain emissions is complicated, other researchers have used more direct methods to apply ambient methods to estimate or constrain NO_*x*_ emissions (e.g., Kourtidis et al., 1999; McDonald et al., 2012; Pierson et al., 1990; Walace et al., 2012). These studies are not subject to many of the limitations of photochemical model evaluations as they have largely relied on near-road measurements to derive emissions estimates rather than trying to rely on photochemical models to interpret measurements made in locations that have been additionally impacted by mixing, transport, chemistry, and deposition.

The source apportionment modeling provided this analysis with the unique ability to track how different emission sources impacted the ΔCO:ΔNO_*y*_ ratio. Our modeling showed that regional transport had a substantial impact on both CO and NO_*y*_ mixing ratios in the Baltimore area. The largest local to regional sources were on-road gasoline, on-road diesel, nonroad, and EGUs. Emitted CO:NO_*y*_ varied by 2 orders of magnitude between sources and spanned the range of measured ΔCO:ΔNO_*y*_ values. This makes connecting an error in modeled ΔCO:ΔNO_*y*_ directly to emission errors from a single source type problematic without further investigation. Comparison between measured and modeled ΔCO:ΔNO_*y*_ showed reasonable agreement in geometric mean values, although measured slopes had substantially more temporal variability than the modeled slopes. Additionally, we demonstrated how both atmospheric processing and entrainment could further impact the calculated ΔCO:ΔNO_*y*_. Modeled ΔCO:ΔNO_*y*_ ratios from mobile source sectors were enhanced 5–50% above emitted CO:NO_*x*_ ratios. Ambient data show a correlation between ΔCO:ΔNO_*y*_ and formaldehyde concentrations that is consistent with the hypothesis that secondary CO formation has increased the ΔCO:ΔNO_*y*_. Together, these analyses suggest that ratios derived from ambient measurements taken during photochemically active conditions may not be directly comparable to emission inventory ratios and should be supplemented with additional analysis. The most favorable conditions for comparison of emission inventory and ambient ratios include times and locations that are (1) predominantly impacted by a single source sector (i.e., power plant plumes, fire plumes, and near-road measurements) and (2) minimally impacted by chemistry and deposition. Comparisons could limit chemistry influences by looking at measurements made very close to the source, in the winter, or at less photochemically active times of day and limit deposition by looking at measurements very close to the source or in elevated plumes.

## Supplementary Material

Supporting Information S1

Data Set S1

## Figures and Tables

**Table 1:** List and Description of Sector Tags Tacked in the Source Apportionment Modeling and Associated CO:NO_*x*_ Ratios and Total CO and NO_*x*_ Emissions

		Emitted molar ratio of CO:NO_*x*_		Emitted domain-wide mass (1,000 tons)	
Tag	Description	Seven counties^a^	Domain wide	CO	NO_*x*_^b^
EGU	Electrical generating units at power plants	0.3	0.5	7	23
NONEGU	Point sources other than power plants	11.5	2.2	12	9
NONPT	Nonpoint area sources	2.9	4.5	10	4
OILGAS	Oil and gas operations (point and area sources)	0.9	1.2	2	3
NONROAD	Nonroad mobile equipment such as construction equipment and lawn and garden equipment	21.9	19.4	222	19
ONGAS	On-road vehicles powered by gasoline	13.8	13.7	199	24
ONDIESL	On-road vehicles powered by diesel fuel	1.0	0.8	10	20
ONOTHER	On-road vehicles powered by other fuels	20.7	21.5	7	0.5
MARINE	Commercial marine vessels (C1, C2, and C3 classes of vessels)	0.1	0.2	1	10
BIOGEN	Biogenic emissions from vegetation and agricultural soil	16.3^c^	20.9^c^	43	3
PTFIRE	Wildland fires (prescribed and wildfires)	102.3	87.5	11	0.2
OTHR	All other emissions sources within the model domain	44.7	63.0	0.5	0.01
BCON	Boundary conditions representing lateral inflow from sources outside of the modeling domain	N/A	N/A	N/A	N/A
ICON	Initial conditions	N/A	N/A	N/A	N/A

*Note*. DISCOVER-AQ = Deriving Information on Surface Conditions from Column and Vertically Resolved Observations Relevant to Air Quality.

a Ratio of CO:NO_*x*_ calculated for emissions in the seven Maryland counties covered by the DISCOVER-AQ flight track: Anne Arundel, Baltimore, Baltimore City, Cecil, Harford, Howard, and Prince George.

b Emitting NO_*x*_ mass is calculated assuming all NO_*x*_ in the form of NO_2_.

c Ratios from the BIOGEN source category include NO emissions from soil in agricultural areas and CO emissions from vegetation (predominantly trees), so this ratio does not represent co-emitted CO and NO_*x*_ from the same sources.

**Table 2:** CO:NO_*y*_ Values for Specific Source Categories at Each Location Investigated in This Study As Well As Emitted CO:NO_*y*_ Values

		EGU	Nonroad	On-road gasoline	On-road diesel
CO:NO_*y*_ ratios in the CMAQ source apportionment modeling	Aldino	0.5 (0.6)	26.8 (28.5)	17.2 (17.5)	**1.5** (1.6)
	Beltsville	0.4 (0.5)	23.4 (27.4)	**15.1 (16.0)**	**1.2 (1.3)**
	Chesapeake Bay	0.6 (0.8)	24.1 (26.4)	16.6 (17.4)	**1.5 (1.9)**
	Edgewood	0.4 (0.5)	26.5 (28.8)	16.8 (17.1)	1.4 (1.6)
	Essex	0.6 (0.6)	28.7 (30.8)	16.3 (16.6)	1.4 (1.5)
	Fairhill	**0.8 (0.9)**	**22.7 (25.2)**	**17.6 (19.1)**	**1.5** (1.7)
	Highway	**0.3 (0.4)**	26.4 (32.4)	15.8 (17.2)	1.3 (1.4)
	Onflight	0.4 (0.5)	24.7 (28.5)	15.8 (16.5)	1.3 (1.4)
	Padonia	0.6 **(0.9)**	**29.9 (33.0)**	16.6 (17.3)	1.4 (1.5)
	All sites average	0.5 (0.6)	25.9 (29.0)	16.4 (17.2)	1.4 (1.5)
CO:NO_*y*_ ratios in the emissions inputs used for the CMAQ modeling	NEI domain wide	0.5 ± 0.7	18.9 ±11.3	13.1 ± 1.1	0.9 ± 0.5
	NEI from seven-county DISCOVER-AQ area	0.3 ± 0.2	21.9 ± 7.1	13.6 ± 0.55	0.9 ± 0.1

*Note*. Main values are derived from ordinary least squares regressions while values in parentheses are derived from orthogonal regressions. The highest and lowest modeled CO:NO_*y*_ value for each source category are shown in bold. NEI ratios were calculated from total emissions in each county. NEI values are mean of county ratios ± standard deviation of ratios among counties. CMAQ = Community Multiscale Air Quality; NEI =National Emission Inventory.

## Key Points:

- Modeling and ambient analysis suggest that atmospheric processing can impact the ΔCO:ΔNO_*y*_ measured by aircraft over the Baltimore region
- For about one third of measurements, NO_*y*_ values derived from different instruments lead to observed ΔCO:ΔNO_*y*_ that are statistically inconsistent
- We urge caution in using ΔCO:ΔNO_*y*_ derived from aircraft measurement to quantify emissions errors from specific sources
