# Supplementary material for: Characterizing CO and NOy Sources and Relative Ambient Ratios in the Baltimore Area Using Ambient Measurements and Source Attribution Modeling
Source: J Geophys Res Atmos. Author manuscript; Available in PMC 2022 Aug 10. (PMC9364951; doi:10.1002/2017jd027688)
Supplement: Supporting Information S1 [file NIHMS1725199-supplement-Supporting_Information_S1.pdf]

Journal of Geophysical Research - Atmospheres

Supporting Information for

Characterizing sources of CO and NOx in the Baltimore area using ambient measurements from the DISCOVER-AQ field study and source attribution modeling

Heather Simon[1], Luke C. Valin[2], Kirk R. Baker[1], Barron H. Henderson[1], James H. Crawford[3], Sally E. Pusede[4], James T. Kelly[1], Kristen M. Foley[2], R. Chris Owen[1], Ronald C. Cohen[5,6], Brian Timin[1], Andrew J. Weinheimer[7], Norm Possiel[1], Chris Misenis[1], Glenn S. Diskin[3], and Alan Fried[8]

1 Office of Air Quality Planning & Standards, US Environmental Protection Agency, RTP, NC

2 National Exposure Research Laboratory, US Environmental Protection Agency, RTP, NC

3NASA Langley Research Center, Hampton, VA

4Department of Environmental Sciences, University of Virginia, Charlottesville, VA

5Department of Chemistry, University of California, Berkeley, Berkeley, CA

6Department of Earth and Planetary Science, University of California, Berkeley, Berkeley, CA

7National Center for Atmospheric Research, Boulder, CO

8 Institute of Arctic and Alpine Research, University of Colorado, Boulder, CO

Contents of this file

Text S1 to S28
Figures S1 to S24
Tables S1 to S2

Introduction

This supplemental information document includes additional tables and figures to provide further details on methods and comparison of NOy and ∑NOy,i measurements , meteorological conditions during the field campaign, CMAQ model evaluation, CMAQ model-predicted source contributions for CO, NO2, and ozone, ΔCO:ΔNOy ratios from all ambient and modeled regressions, and estimation instantaneous ΔCO:ΔNOy ratios.

Text S1. Description of measurement methods and comparison of NOy and ∑NOy,i measurements

CO was measured on the NASA P-3B aircraft by the DACOM (Differential Absorption CO Measurements) instrument (Sachse, et al., 1987). Ambient air was supplied to the instrument via a Rosemont probe inlet and an

inline compressor at a nominal flowrate of 5 slpm. Measurements of CO (as well as CH4 and N2O) are made using a wavelength-modulated mid-infrared diode laser which is passed through the measurement volume in a reduced-pressure astigmatic Herriott multipass cell. Nominal temporal response of the instrument is 1 Hz, with 1% precision. Calibration gases, assayed by NOAA/ESRL, are introduced into the system periodically to maintain accuracy of the measurements at 2%.

Formaldehyde (CH_2O was measured on the NASA P3B aircraft using the DFGAS (Difference Frequency Generation Absorption Spectrometer) instrument, comprehensive details for which can be found in Fried et al (2016) and references therein. The measurement principle is similar to DACOM, but the DFGAS instrument employed a more sophisticated mid-IR laser source based upon difference frequency mixing of two near-IR lasers. As discussed, DFGAS provided CH_2O data with 1–2 s time resolution with limits of detection (1σ LOD) in the 47 to 66 pptv range, with most values falling the 50 – 60 pptv range at 1 second time resolution. The 1 minute LOD improved to around 20 pptv, and the estimated accuracy in all cases is ~ 4%.

NO, NO2, and NOy, and O3 were measured with the NCAR 4-channel chemiluminescence instrument on board the P3B aircraft. For the NO channel, reagent O3 is generated and mixed with the sample flow resulting in the chemiluminescent reaction that creates excited NO2 molecules in proportion to ambient NO. The resulting photons are counted with a dry-ice-cooled photomultiplier tube. NO2 is measured by converting a large fraction of the NO2 to NO in a photolytic converter in a separate sample flow, followed by detection as NO. The signal due to ambient NO is subtracted and an adjustment is made for the sub-unity conversion efficiency to NO. NOy is measured in a third sample flow by catalytically converting NOy species to NO in a gold-tube converter heated to 300 C. The sensitivity of all channels to NO is measured periodically during flight by adding a small flow from a calibration standard with a known mixing ratio of NO in N2. The NO2 conversion efficiencies of the NO2 and NOy converters are also measured as part of the calibration sequence by converting a large, measured fraction of the calibration NO to NO2 by reaction with O3 prior to addition to the sample flow. Other than for periodic sensitivity, zero, and artifact determinations, data are recorded continuously and reported at 1 s with nominal uncertainty of 10% for NO measurements, 15% for NO_2, 20% for NO_y and 5% for O_3.

NO2 was also measured by laser-induced fluorescence (LIF) (Thornton et al., 1999). This instrument uses a Q-switched, frequency doubled Nd3+:YAG laser to pump a tunable dye laser, which is etalon-tuned between a specific 585 nm rovibronic NO2 feature and the background continuum absorption. The resulting red-shifted photons are collected with a photomultiplier tube using time-gated counting. LIF data are selective for NO2 and accurate to ±5%, with the system calibrated at least every 30 min in flight with an NO2 reference added at the inlet. To observe the atmospheric products of NOx, thermal dissociation (TD) is coupled to LIF. Peroxy nitrates (PNs; ΣRO2NO2), alkyl nitrates (ANs; ΣRONO2), and nitric acid (HNO3) each dissociate into NO2, detected by LIF, and a companion radical at characteristic temperatures of greater than 220 ºC, 380 ºC, and 650 ºC, respectively. Mixing ratios of each are then determined as the difference between heated channels. For example, ANs are measured as the difference between the 380 ºC channel (ANs + PNs + NO2) and the 220ºC channel (PNs + NO2). Accuracy for the higher oxide measurements includes terms for the completeness of dissociation to NO2 and the efficiency of transmission through the inlet. Accuracy is estimated for this DISCOVER-AQ deployment according to Day et al. (2002) to be ±10% for PNs and ±15% for ANs and HNO3. HNO3 measurements represent both gas-phase HNO3 and aerosol-phase nitrate in particles smaller than PM2.5 (Pusede et al., 2016). The TD-LIF instrument used here is a two-cell system. Data were collected at 4 Hz and averaged to 1 second, such that measurements were made in the following cycle: NO2 and PNs (8 s), ANs (8 s), NO2 + PNs (8 s), and HNO3 (8 s), with 6 off-line seconds between each species sampling period. In order to better characterize measurement uncertainty of NOy and ∑NOy,I, we compare these two measurements matched in space and time for measurements taken within the boundary layer on each flight day in Figure S1. While the best efforts were made

to match measurements in time, it should be noted that since ∑NOy,I is calculated by summing NOy components that were measured up to 2.5 minutes apart. In contrast the NOy measurements were aggregated to 15 second averages. Therefore, the ∑NOy,I values represent slightly longer time averages which may impact comparisons at times during the flight when NOy is changing rapidly, such as during spirals. While the comparisons generally line up close to the 1:1 line in figure S1, there are flight to flight differences in these two measurement methods for NOy. Measurements made on July 1, 2, 5, 16 and 20 generally fall slightly above the 1:10 line, meaning that ∑NOy,I was consistently higher than measured NOy on those flights. Conversely, data points fall consistently below the 1:1 line on July 10, 11, 14, 21, 22, 26, 27, 28, and 29 meaning that ∑NOy,I was consistently higher than NOy on those flight days.

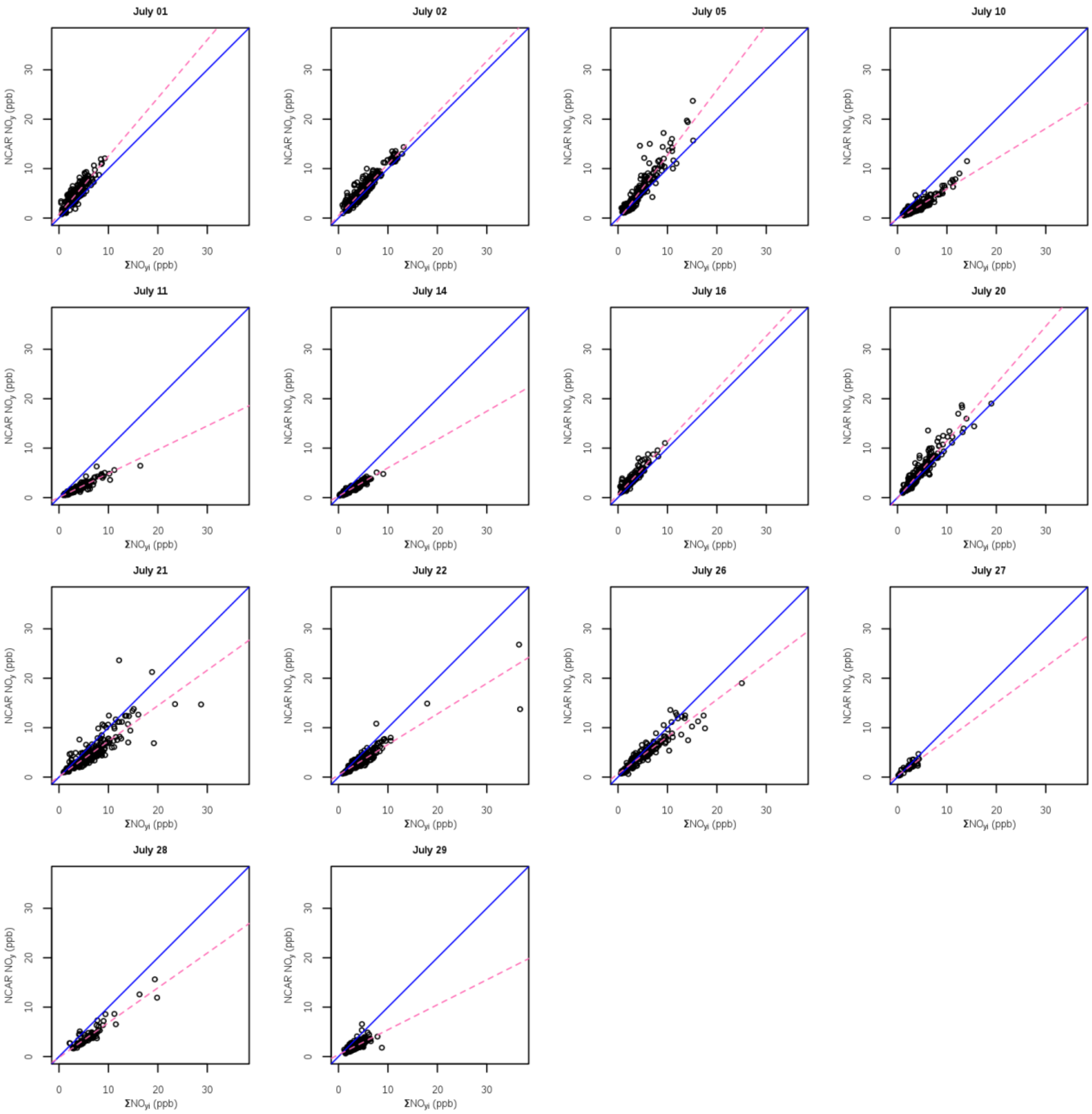

Figure S1: comparison of NOy measured by NCAR with ∑NOy,i measurements on each P3B flight. 1:1 line shown in blue. Pink dashed line shows linear regression of NOy versus ∑NOy,i.

Figure S2 shows a time series of the difference between measured ∑NOy,i and NOy on each flight. The differences appear to periodically get larger and then smaller, with larger differences corresponding to higher NOy mixing ratios which occur lower in the boundary layer (Figures S3 and S4). The periodic increases and decreases in differences therefore correspond to ascents and descents of the P3B aircraft. On most days (with the exception of Jul 20, 21, and 22) there is little visible progression of this difference over the course of a flight. In other words, the difference between measurement methods does not get substantially larger or smaller as the flight progresses indicating that these differences are not due to any failure to correct for instrument drift.

To further investigate the drivers of the differences between the two NOy measurement methods, we compare the difference between them with corresponding mixing ratios of each NOy components (Figures S3-S10). On flights where ∑NOy,i was consistently larger than NOy, we see positive correlation between the differences in the measurements with total NOy and each NOy species (NO, NO2, HNO3, ANs, PNs). Conversely, on days where ∑NOy,i was consistently smaller than NOy there is a consistent negative correlation with total NOy and NO and NO2. However, on those days, there is little to no correlation between the measurement difference and the NOz species (HNO3, AN, PN). This suggests that on flights where ∑NOy,i was greater than NOy the discrepancy does not appear to be due to any particular species, but on flights were ∑NOy,i was less than NOy the discrepancy appears to be related to NO and NO2, but not related to aged NOz species.

Finally, in order to better understand how these measurement differences relate to estimated CMAQ model biases in NOy, we compare differences between ∑NOy,i and NOy with model bias using NOy measurements (Figure S9) and model bias using ∑NOy,i (Figure S10). The model bias has moderate correlation (or anti-correlation) (magnitude of r between 0.25 and 0.63) with measurement differences on 8 of the 14 flight days for each of the two NOy measurement methods, with very low correlation on the other 6 flight days. For days where ∑NOy,i was greater than NOy, comparing the modeled NOy with ∑NOy,i generally improved model over-predictions seen when modeled NOy was compared with measured NOy. On those days, model mean bias ranged from -1.6 ppb to 7.1 ppb when calculated with measured NOy and ranged from -2.3 ppb to 5.5 ppb when calculated with ∑NOy,i. on days where ∑NOy,i was smaller than NOy, the impact of the NOy measurement method on model performance was mixed, although in general model NOy performance was better on those 5 days (-0.1 ppb to 2.6 ppb).

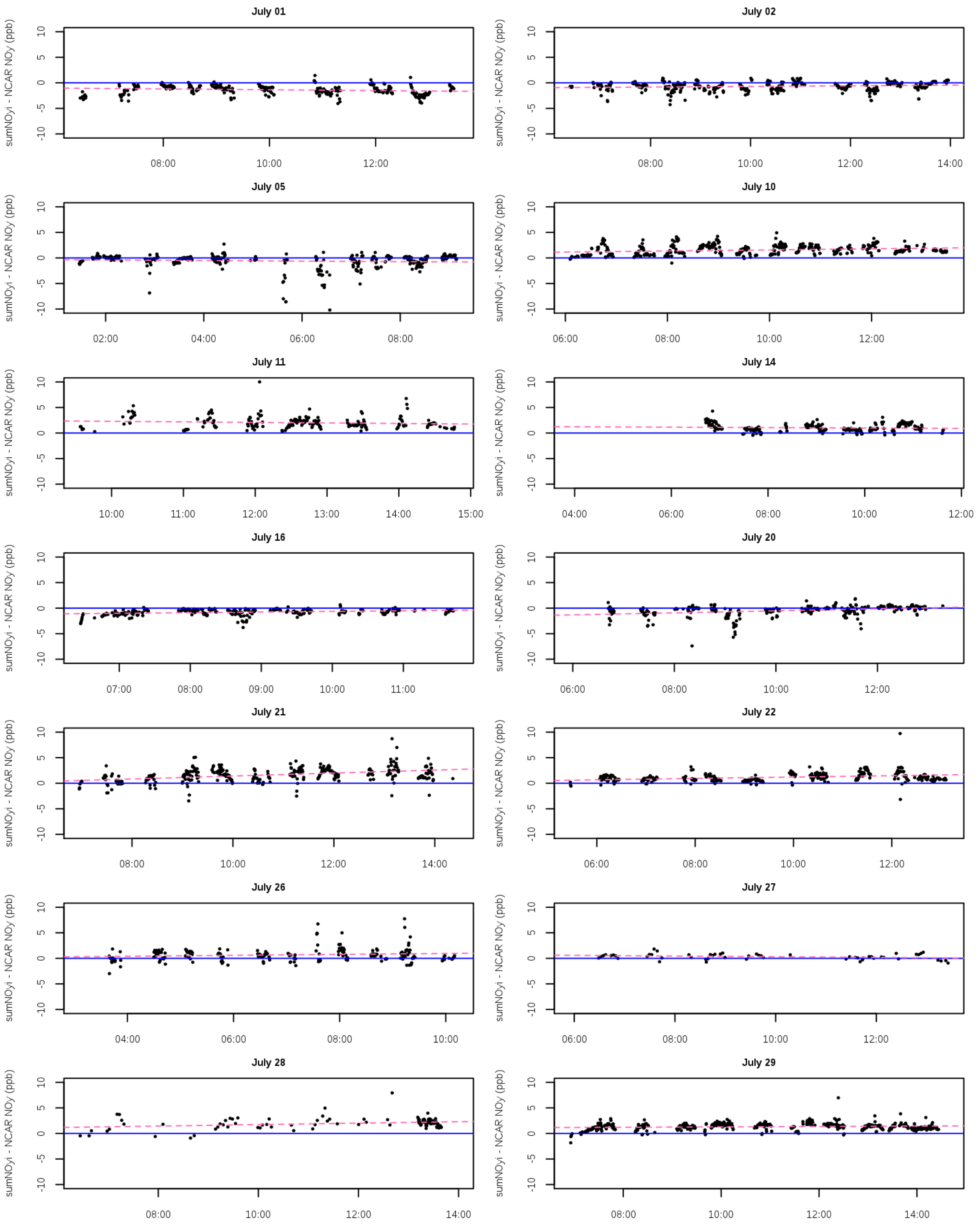

Figure S2: Time series of differences between ∑NOy,i and NOy measurements on each flight day. Blue line shows zero difference. Pink dashed line shows the linear regression of this difference over time for each flight.

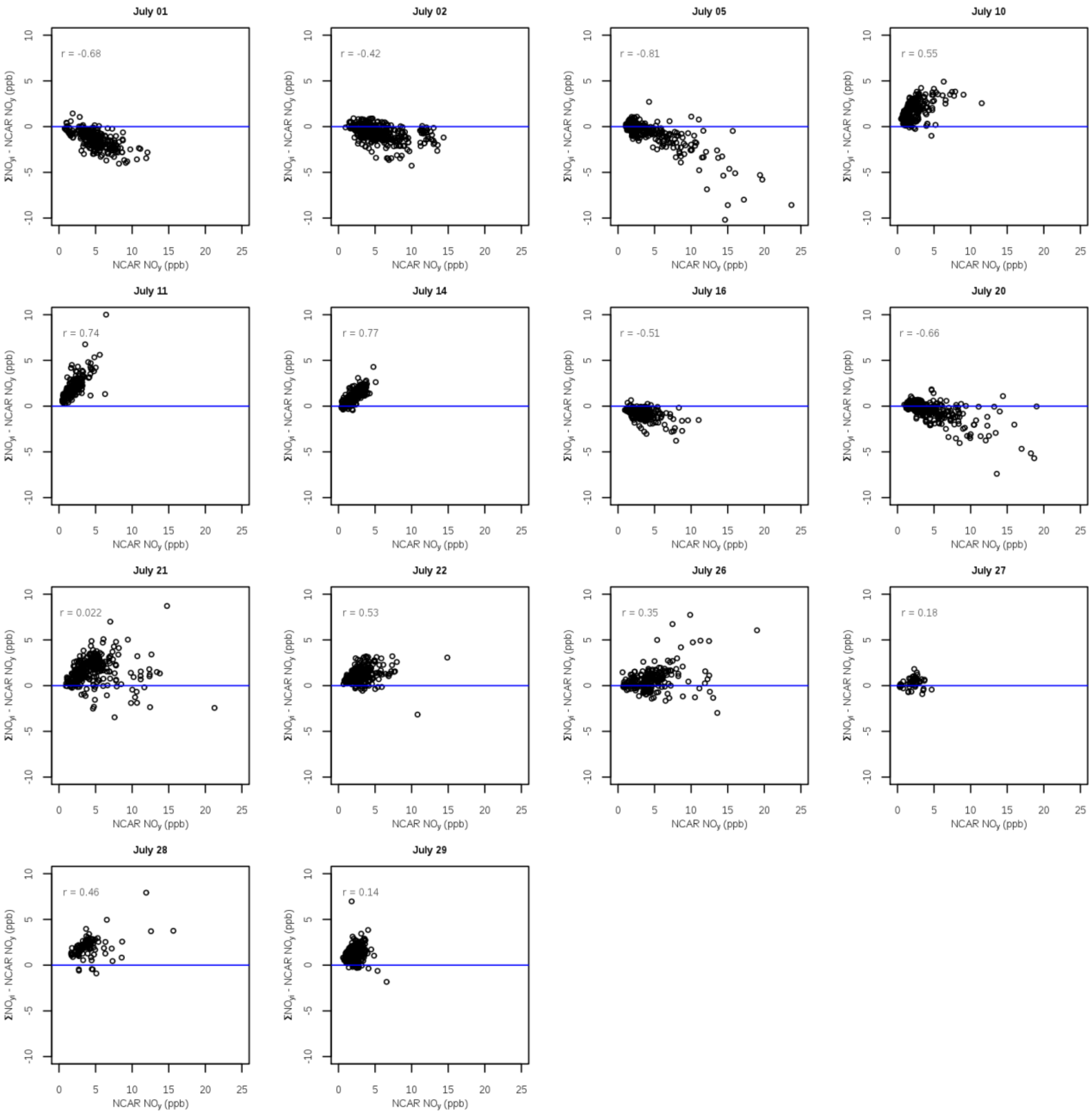

Figure S3: Differences between ∑NOy,i and NOy compared to total measured NOy on each flight day. Blue lines indicate zero difference. Correlation values (r) between the NOy measurement differences and total NOy are given for each flight day.

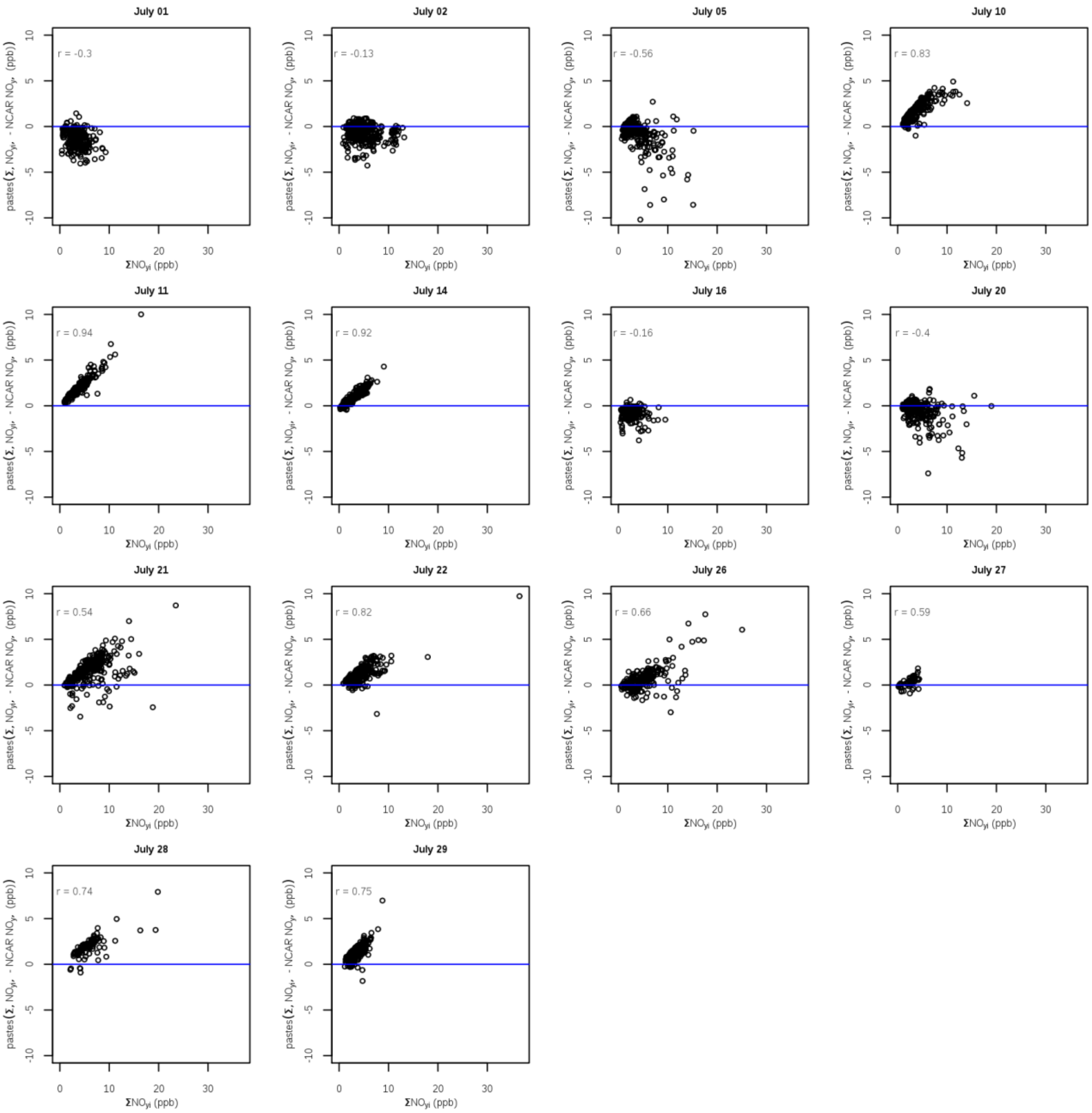

Figure S4: Differences between $\sum NO_{y,i}$ and NO_y compared to $\sum NO_{y,i}$ on each flight day. Blue lines indicate zero difference. Correlation values (r) between the NO_y measurement differences and $\sum NO_{y,i}$ are given for each flight day.

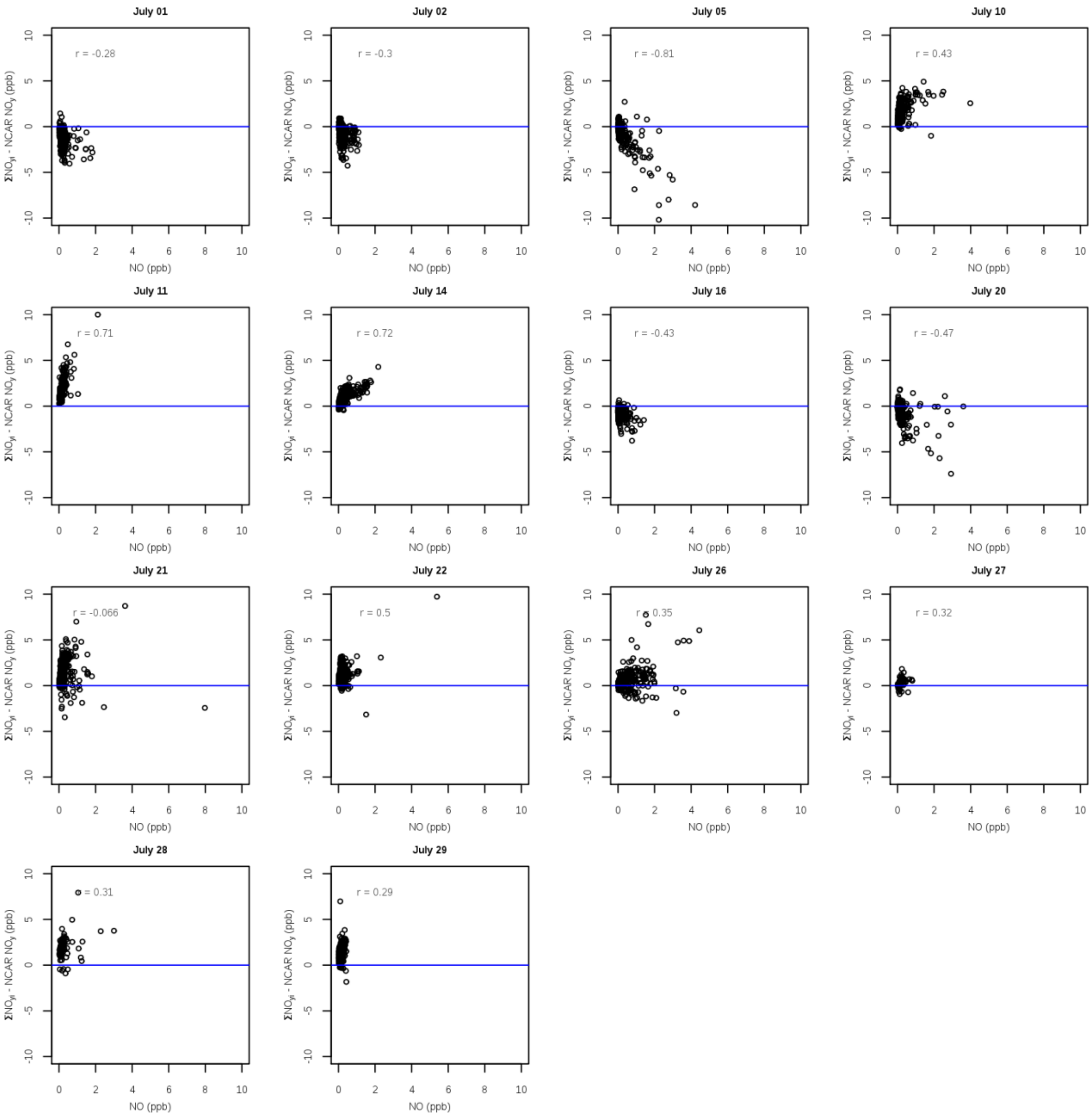

Figure S5: Differences between ∑NOy,i and NOy compared to measured NO on each flight day. Blue lines indicate zero difference. Correlation values (r) between the NOy measurement differences and NO are given for each flight day.

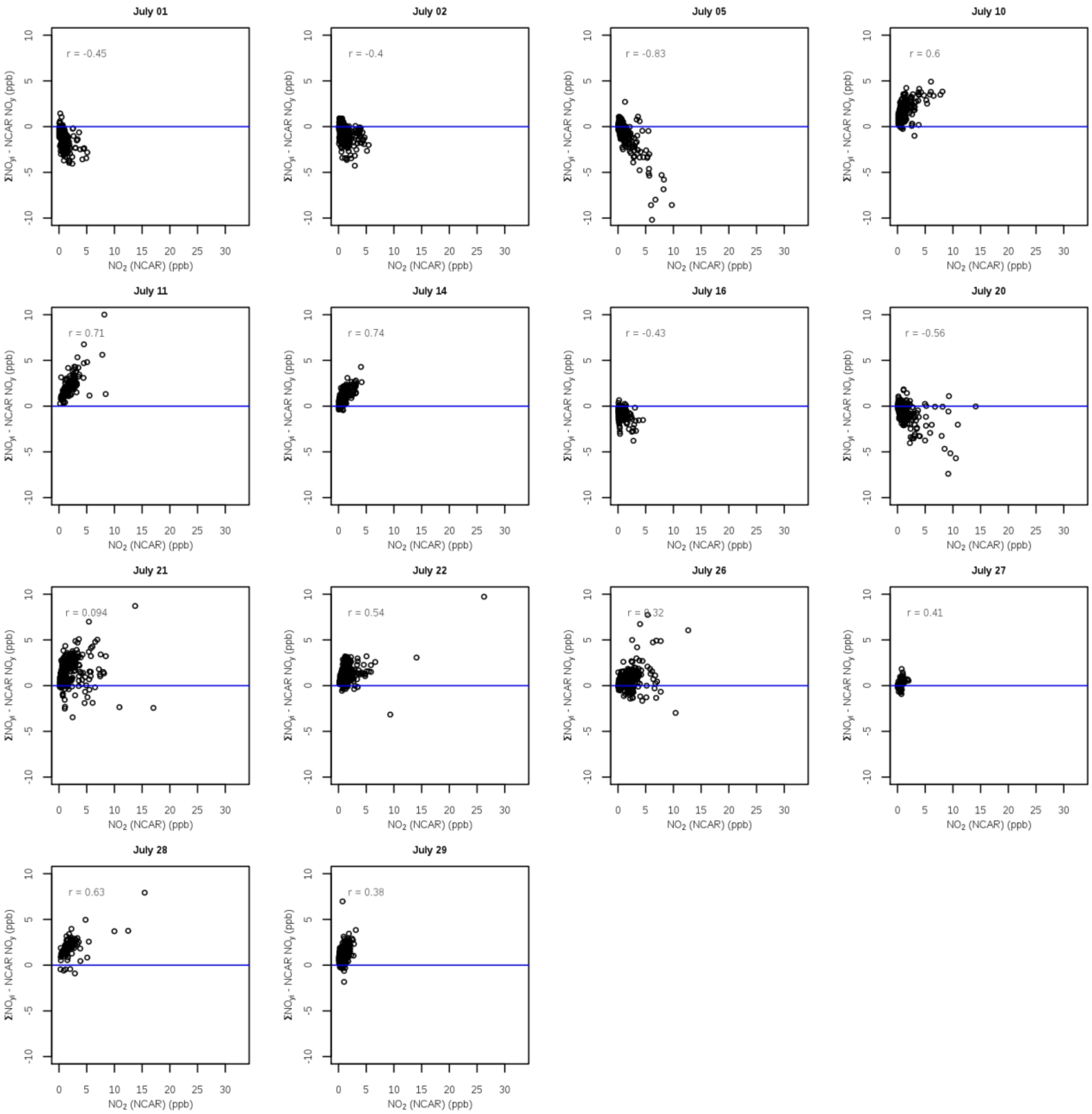

Figure S6: Differences between ∑NOy,i and NOy compared to measured NCAR NO2 on each flight day. Blue lines indicate zero difference. Correlation values (r) between the NOy measurement differences and NCAR measured NO2 are given for each flight day.

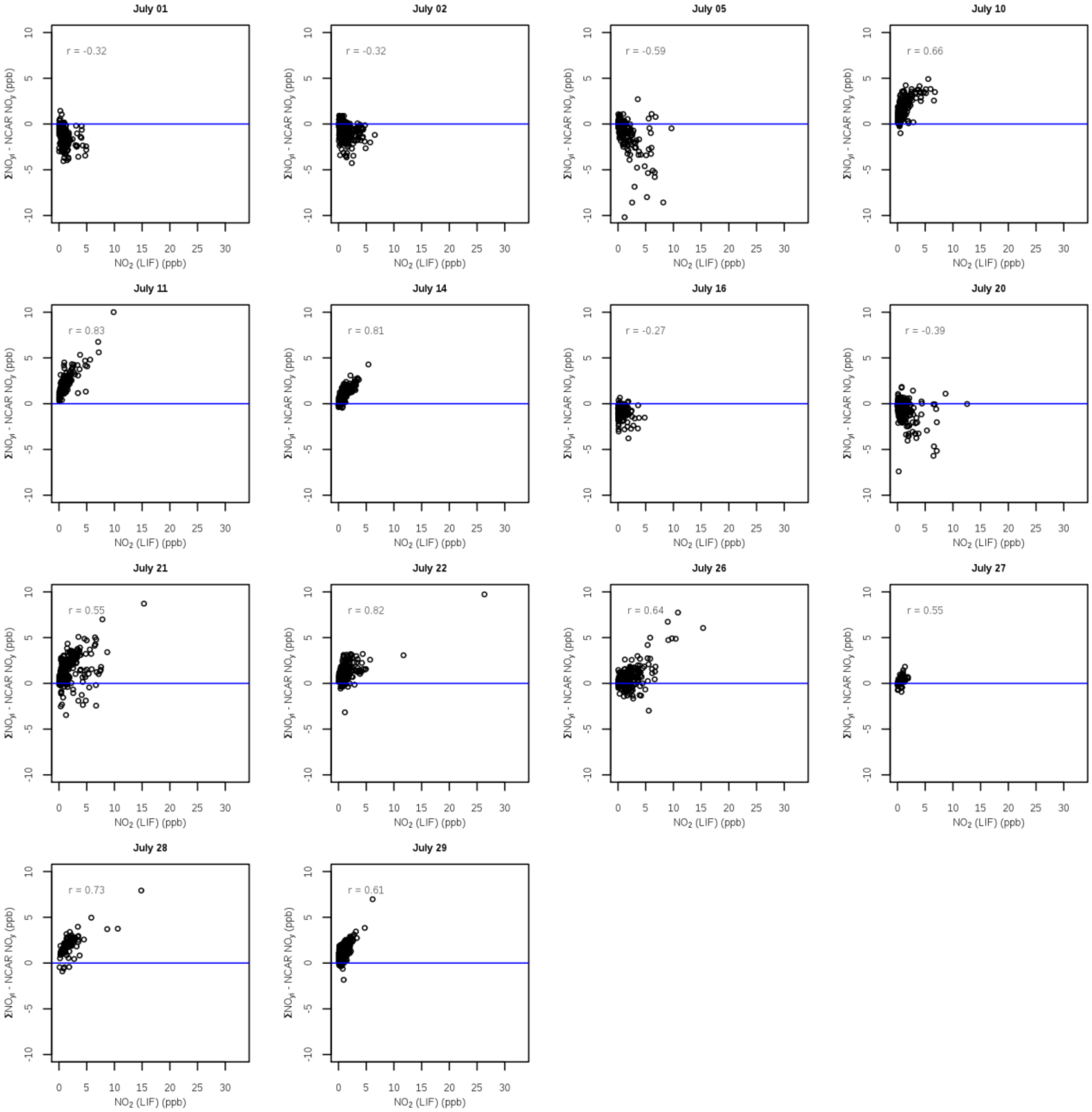

Figure S7: Differences between $\sum NO_{y,i}$ and NO_y compared to measured LIF NO_2 on each flight day. Blue lines indicate zero difference. Correlation values (r) between the NO_y measurement differences and LIF measured NO_2 are given for each flight day.

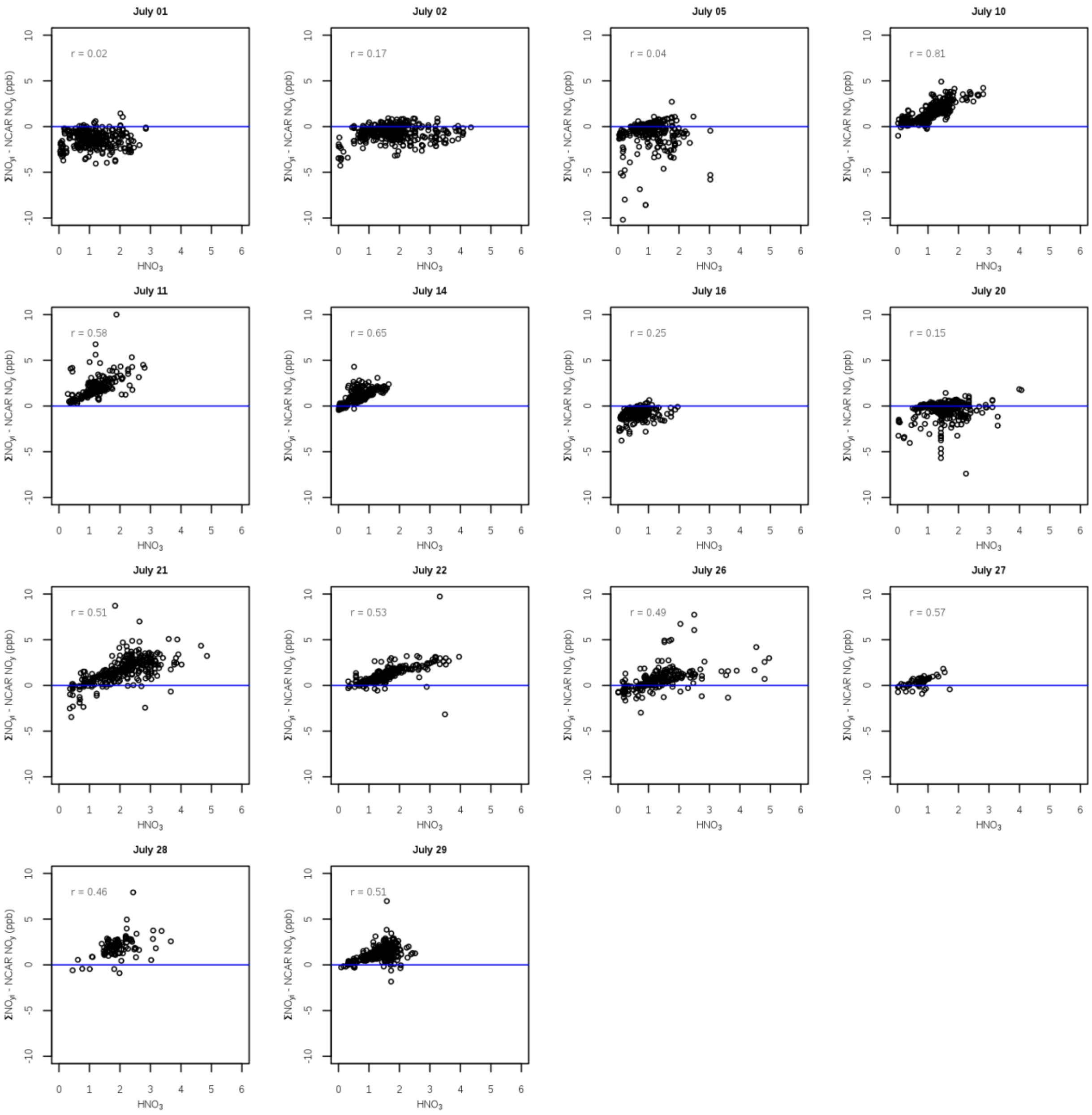

Figure S8: Differences between ∑NOy,i and NOy compared to measured HNO3 on each flight day. Blue lines indicate zero difference. Correlation values (r) between the NOy measurement differences and HNO3 are given for each flight day.

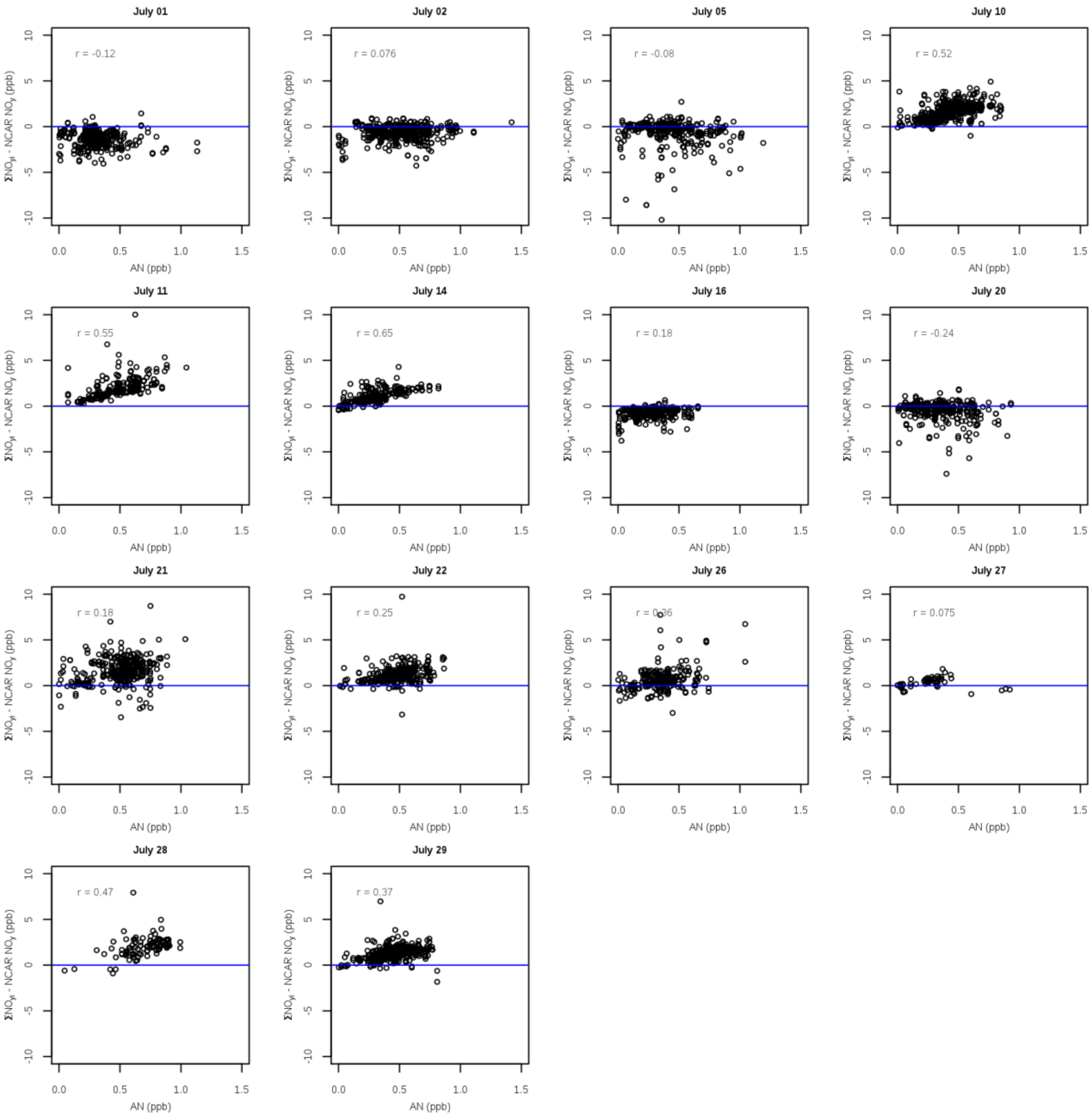

Figure S9: Differences between $\sum NO_{y,i}$ and NO_y compared to measured ANs on each flight day. Blue lines indicate zero difference. Correlation values (r) between the NO_y measurement differences and AN are given for each flight day.

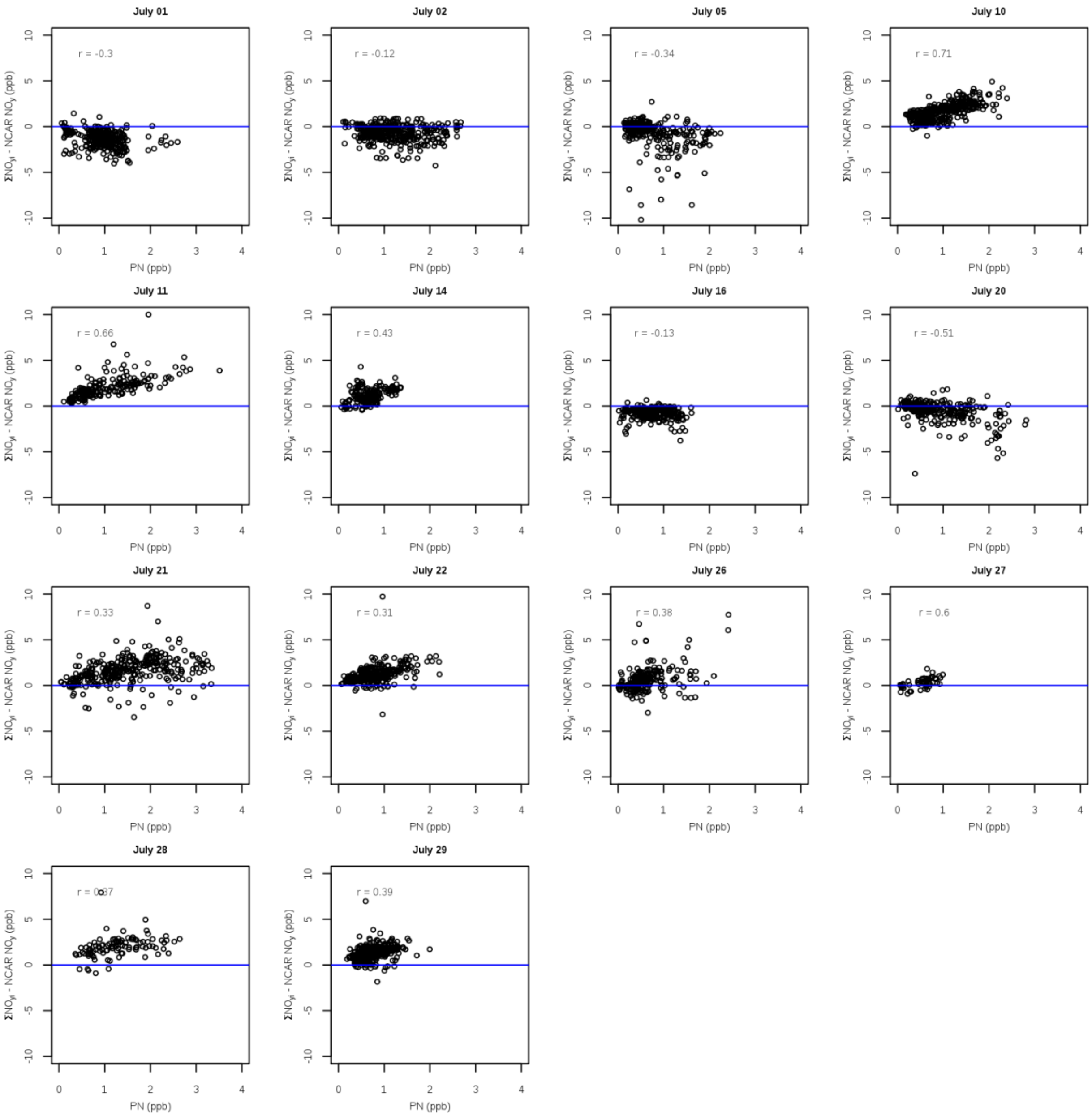

Figure S10: Differences between ∑NOy,i and NOy compared to measured PNs on each flight day. Blue lines indicate zero difference. Correlation values (r) between the NOy measurement differences and PN are given for each flight day.

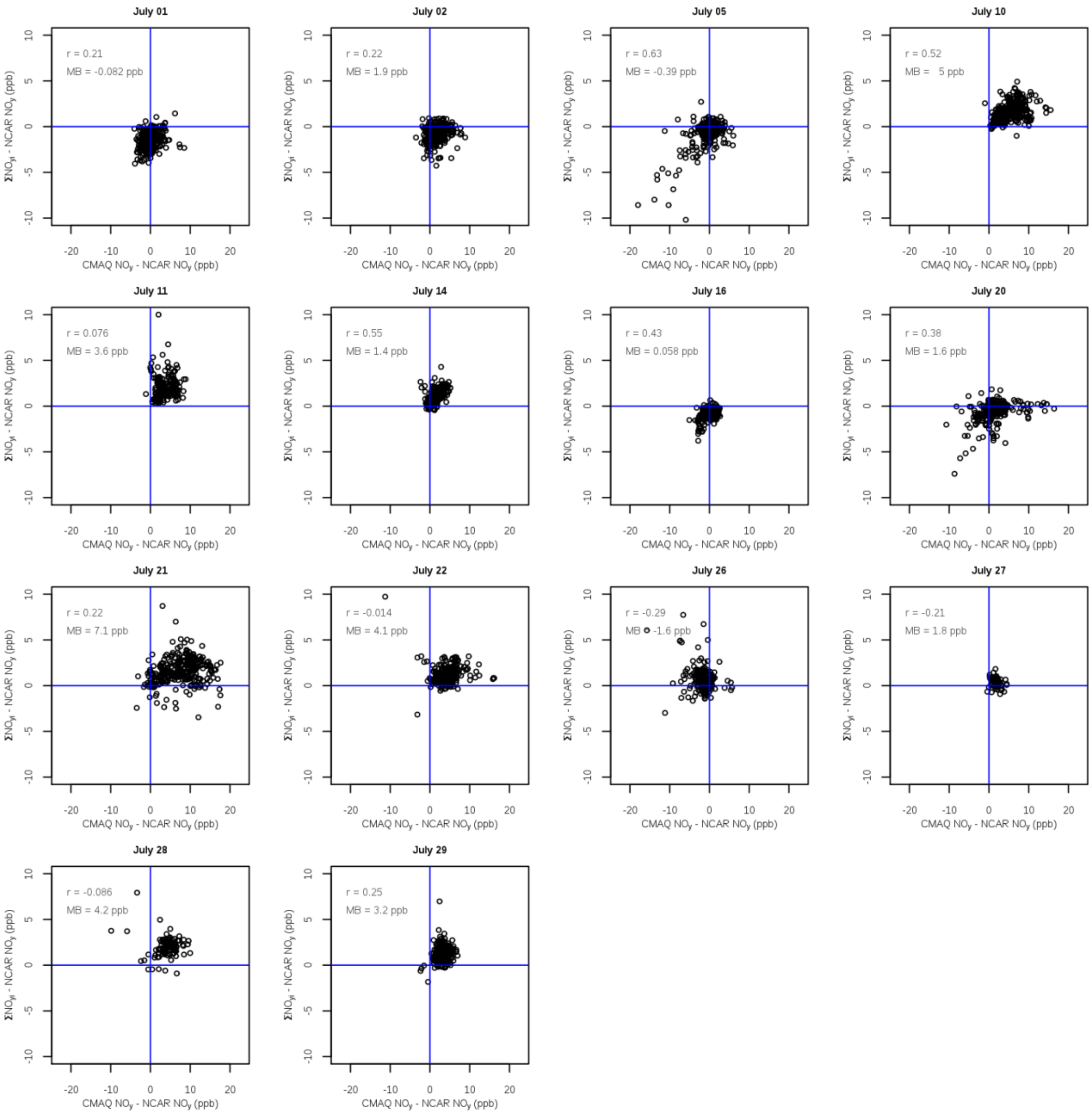

Figure S11: Differences between ∑NOy,i and NOy compared to CMAQ model bias using NOy on each flight day. Blue lines indicate zero difference and zero CMAQ model bias. Correlation values (r) between the NOy measurement differences and measured NOy are given for each flight day. In addition, mean bais (model NOy – measured NOy) are also given for each flight day.

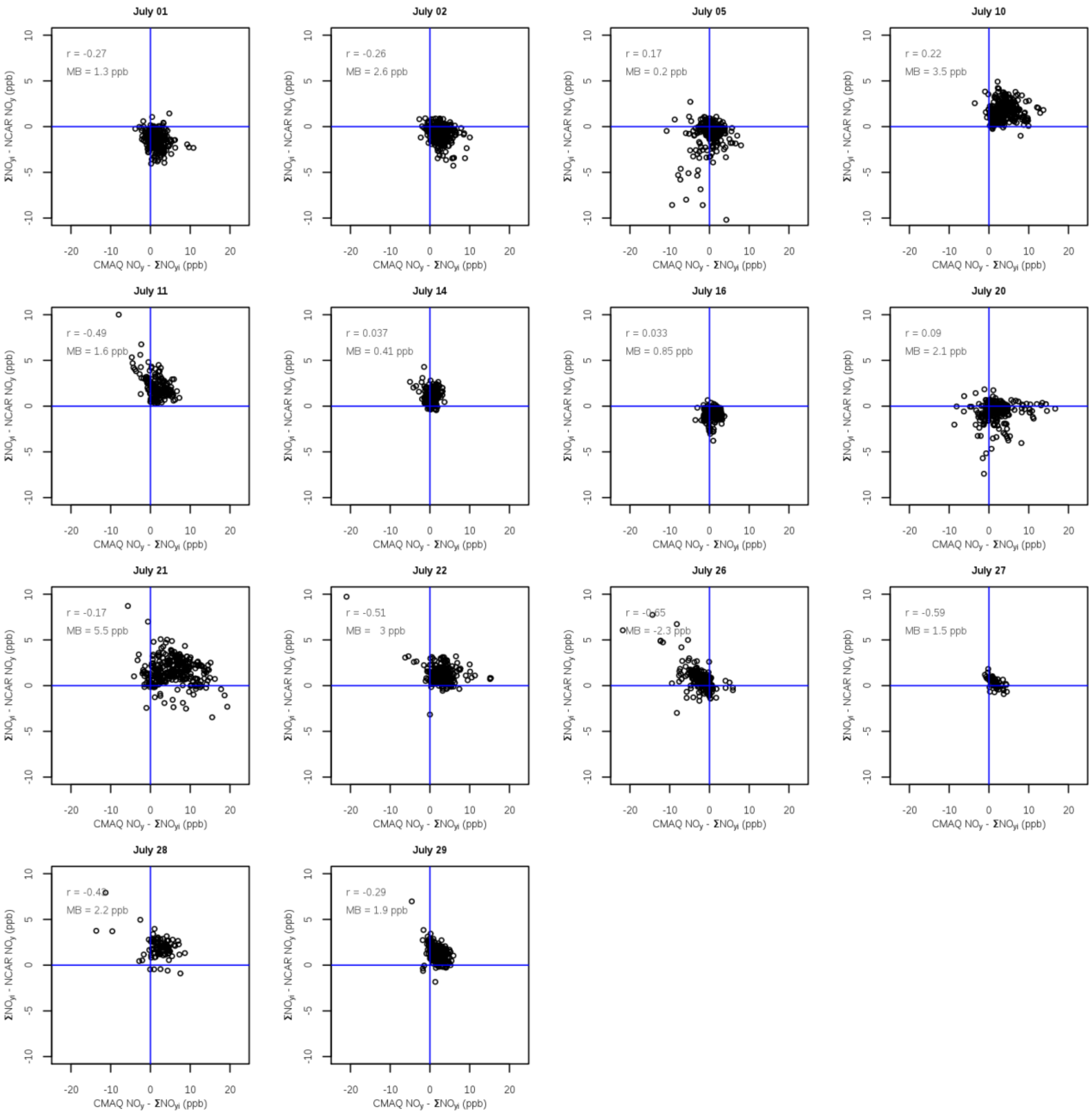

Figure S12: Differences between ∑NOy,i and NOy compared to CMAQ model bias using ∑NOy,i on each flight day. Blue lines indicate zero difference and zero CMAQ model bias. Correlation values (r) between the NOy measurement differences and ∑NOy,I are given for each flight day. In addition, mean bais (model NOy – ∑NOy,i) are also given for each flight day.

Section S2: Meteorological information

Table S1: Summary of July 2011 meteorological conditions in Baltimore, MD

Date	Day	Max Temp (F)	Max RH (%)	Min Visibility (Miles)	Max Wind Speed (MPH)	Mean Wind Speed (MPH)	Precipitation (Inches)	Cloud Cover	Events*	Flight Day?
**6/30/2011**	Thu	86	84	10	15	3	0	2		N
**7/1/2011**	Fri	89	84	10	12	3	0	1		Y
**7/2/2011**	Sat	93	90	10	14	3	0	3		Y
**7/3/2011**	Sun	93	93	1	28	6	0.55	7	R,T	N
**7/4/2011**	Mon	89	93	9	7	3	T	7		N
**7/5/2011**	Tue	93	90	6	12	3	0	3		Y
**7/6/2011**	Wed	89	93	5	12	4	T	5	R	N
**7/7/2011**	Thu	95	100	2	30	5	0.1	3	R,T	N
**7/8/2011**	Fri	87	93	0	22	4	0.83	7	F,R,T	N
**7/9/2011**	Sat	90	97	5	14	5	0	3		N
**7/10/2011**	Sun	90	84	7	15	3	0	2		Y
**7/11/2011**	Mon	93	94	2	31	8	0.17	4	R,T	Y
**7/12/2011**	Tue	93	94	9	17	8	0	5		N
**7/13/2011**	Wed	92	93	2	15	4	0.26	5	R,T	N
**7/14/2011**	Thu	83	68	10	16	6	0	3		Y
**7/15/2011**	Fri	83	87	10	13	4	0	3		N
**7/16/2011**	Sat	87	90	9	15	5	0	2		Y
**7/17/2011**	Sun	91	78	10	13	5	0	3		N
**7/18/2011**	Mon	95	90	9	14	6	0	4		N
**7/19/2011**	Tue	95	85	1	16	4	0.16	7	R,T	N
**7/20/2011**	Wed	93	93	4	10	4	0	3		Y
**7/21/2011**	Thu	100	94	2	15	5	0	2	F	Y
**7/22/2011**	Fri	106	85	5	16	5	0	3		Y
**7/23/2011**	Sat	102	79	7	15	5	0.06	4	R	N
**7/24/2011**	Sun	98	85	6	14	7	0	5		N
**7/25/2011**	Mon	91	94	1	14	4	0.64	6	R,T	N
**7/26/2011**	Tue	95	93	2	14	5	0	2	F	Y
**7/27/2011**	Wed	90	73	10	15	5	0	4		Y
**7/28/2011**	Thu	91	84	10	9	3	T	6		Y
**7/29/2011**	Fri	101	85	5	21	6	0	3		Y
**7/30/2011**	Sat	96	82	9	16	5	0	4		N

*Rain (R), Thunderstorms (T), Fog (F)

Section S3: Additional Model Performance Analysis

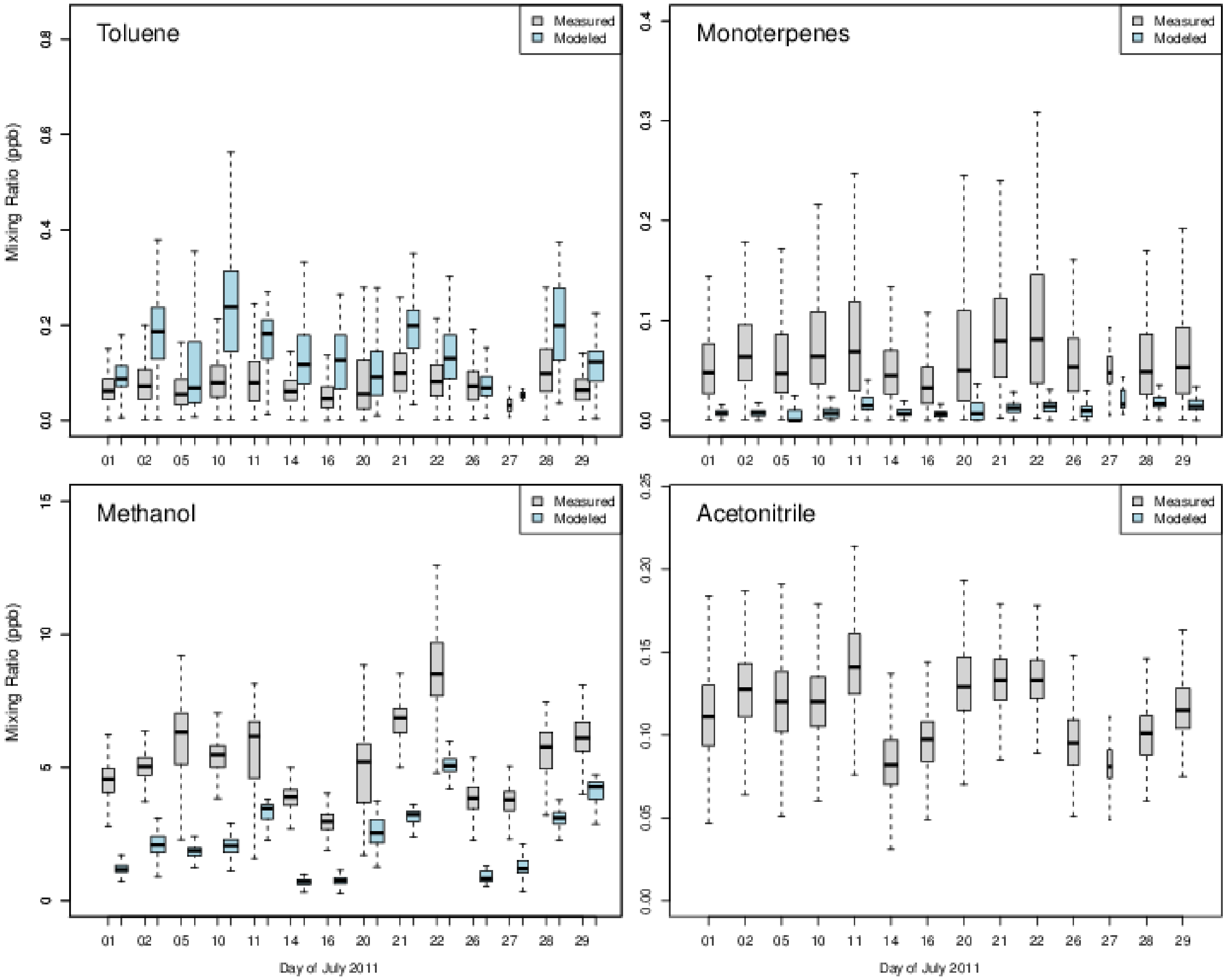

Figure S13. Modeled and observed mixing ratios of organic species (toluene, methanol, monoterpenes, acetonitrile) from the P3B aircraft.

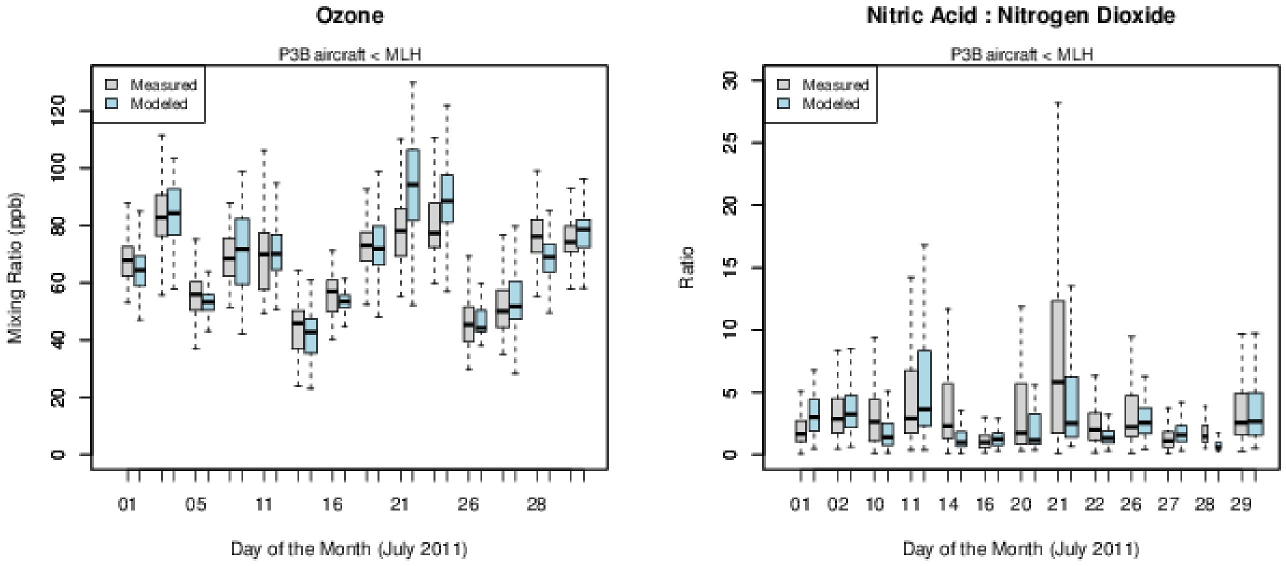

Figure S14. Measured and modeled mixing ratios of Ozone and HNO_3:NO_2 from the P3B aircraft.

The modeling system does well at capturing the magnitude and spatial variability in O3 over the Chesapeake Bay compared to ship based measurements from this field study (Figure S15). Nitrogen oxide peak measurements are captured by the model but the modeling system consistently had high predictions even where measurements showed low values. Source apportionment modeling indicates fairly similar contribution from the commercial marine sector, local to regional anthropogenic sources, and biogenics to O3 over the Chesapeake Bay. However, the commercial marine sector tended to contribute most to NO, especially nearer to the Port of Baltimore. The model does estimate notable local to regional anthropogenic source contribution to NO in the Chesapeake Bay on certain days when meteorological conditions are favorable.

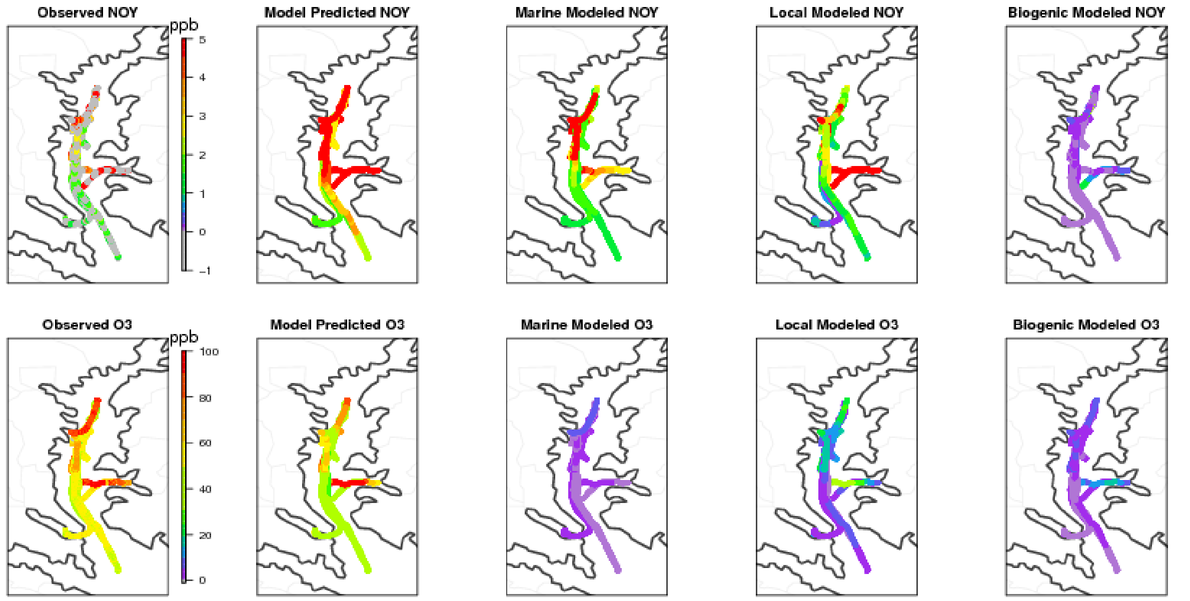

Figures S15: Observed and modeled O3 and NO from ship measurements taken from the Chesapeake Bay on July 11-18 and July 20, 2011.

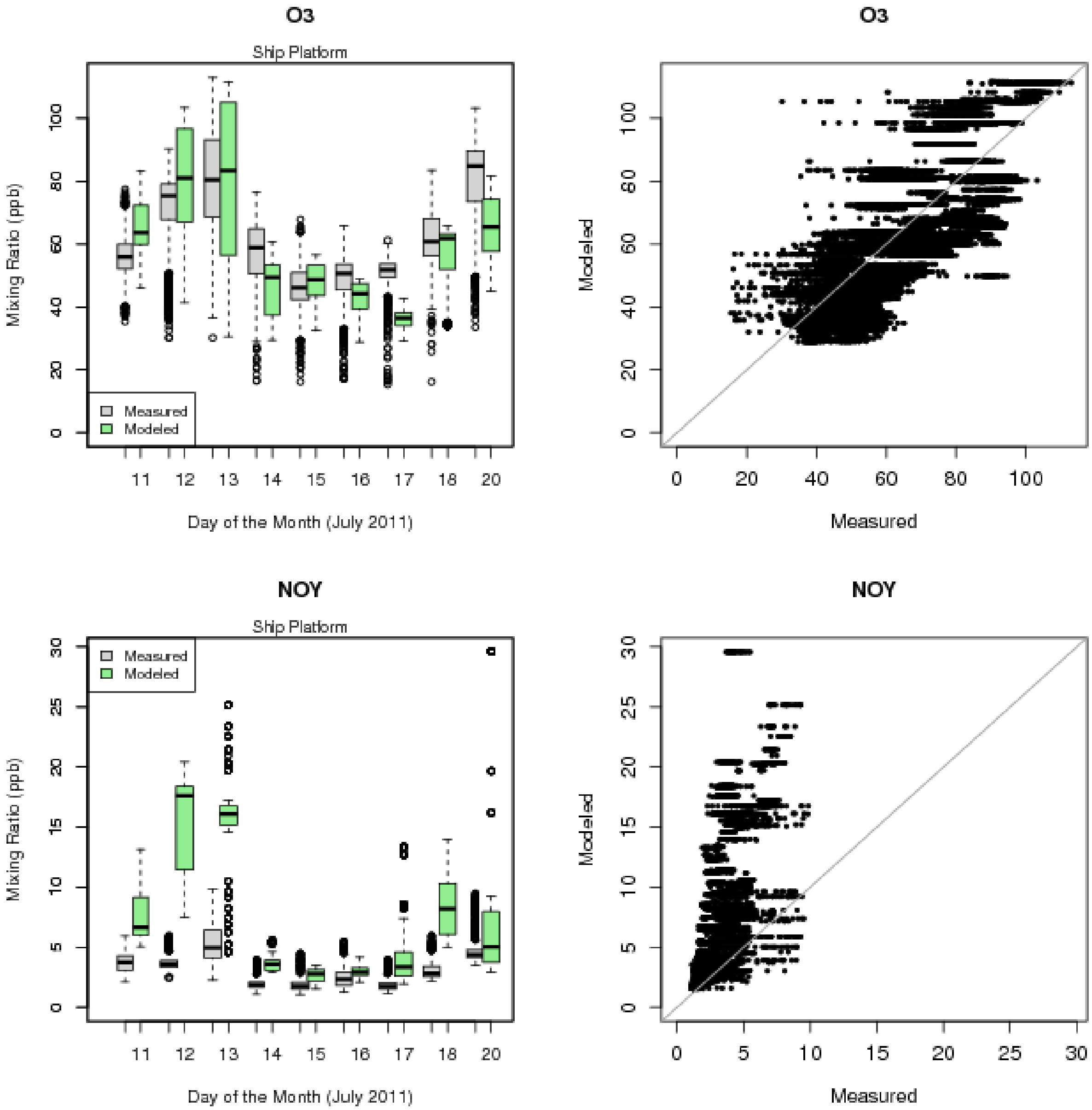

Figure S16: Distribution of O3 and NO from ship measurements (and paired model predictions) taken from the Chesapeake Bay on July 11-18 and July 20, 2011.

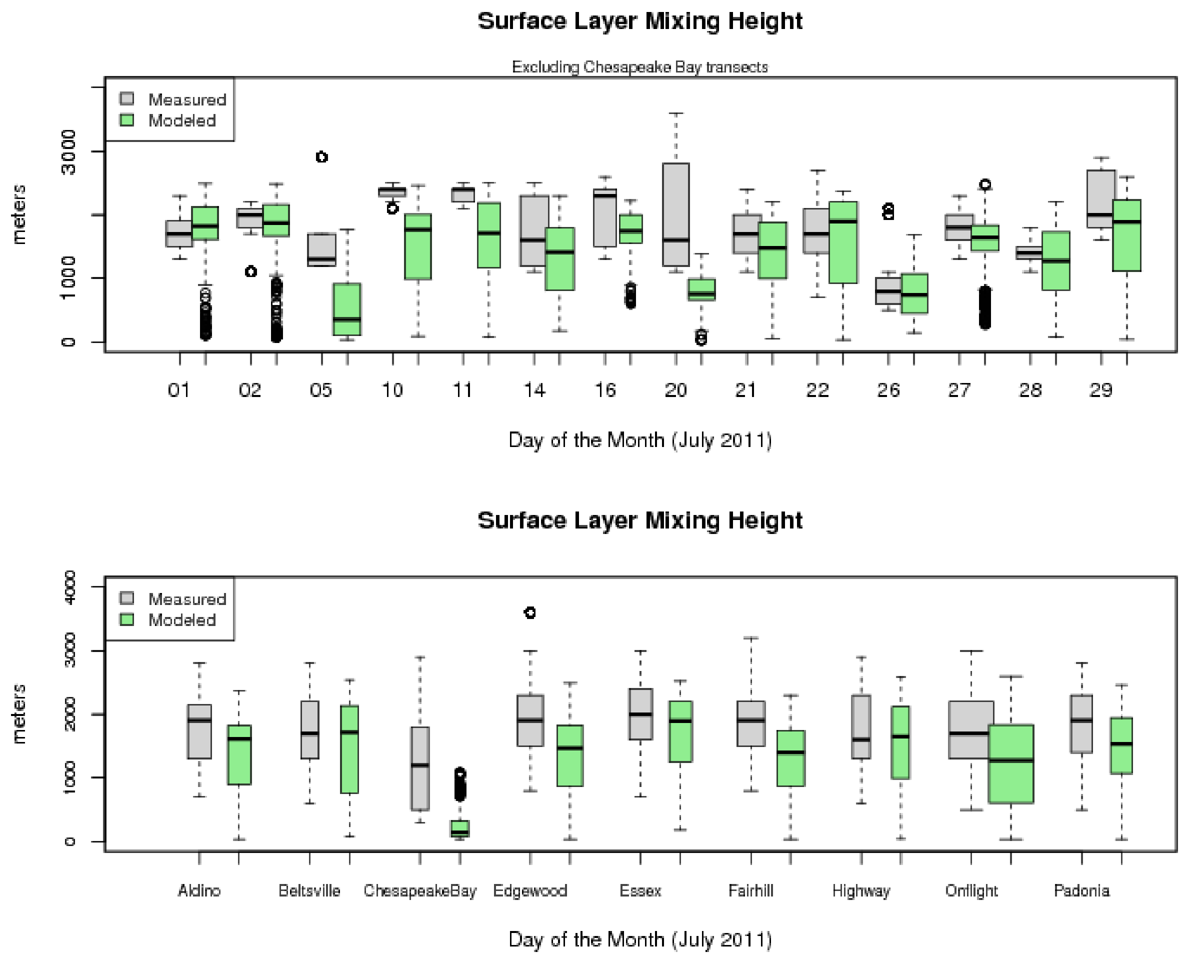

Figure S17: Modeled and measured mixed layer heights by flight day and location.

Section S4: O3, CO, and NO2 modeled source contributions

Figure S18 shows the distribution of sector contribution to model estimated O3, CO, and NO2 at times and locations that match aircraft measurements below 2 km in altitude. The largest contributing sectors to aircraft O3 include fairly similar amounts from biogenics, EGUs (local to regional), nonroad, onroad diesel, and onroad gasoline sources. The largest contributions to modeled CO include fairly comparable amounts from onroad gasoline and nonroad (gasoline) sources. For NO2, the largest contributing sectors include onroad gasoline, onroad diesel, nonroad, and EGUs (local to regional) which represent a different mix than seen for either CO or O3 for this area. If these sector contributions are similar for the ambient data then ratios of CO and NO2 or NOY may not truly represent any specific sector at the time and locations of these aircraft measurements. Figures S19 and S20 show spatial maps of the July 2011 average contributions of each source category to CO and ozone respectively.

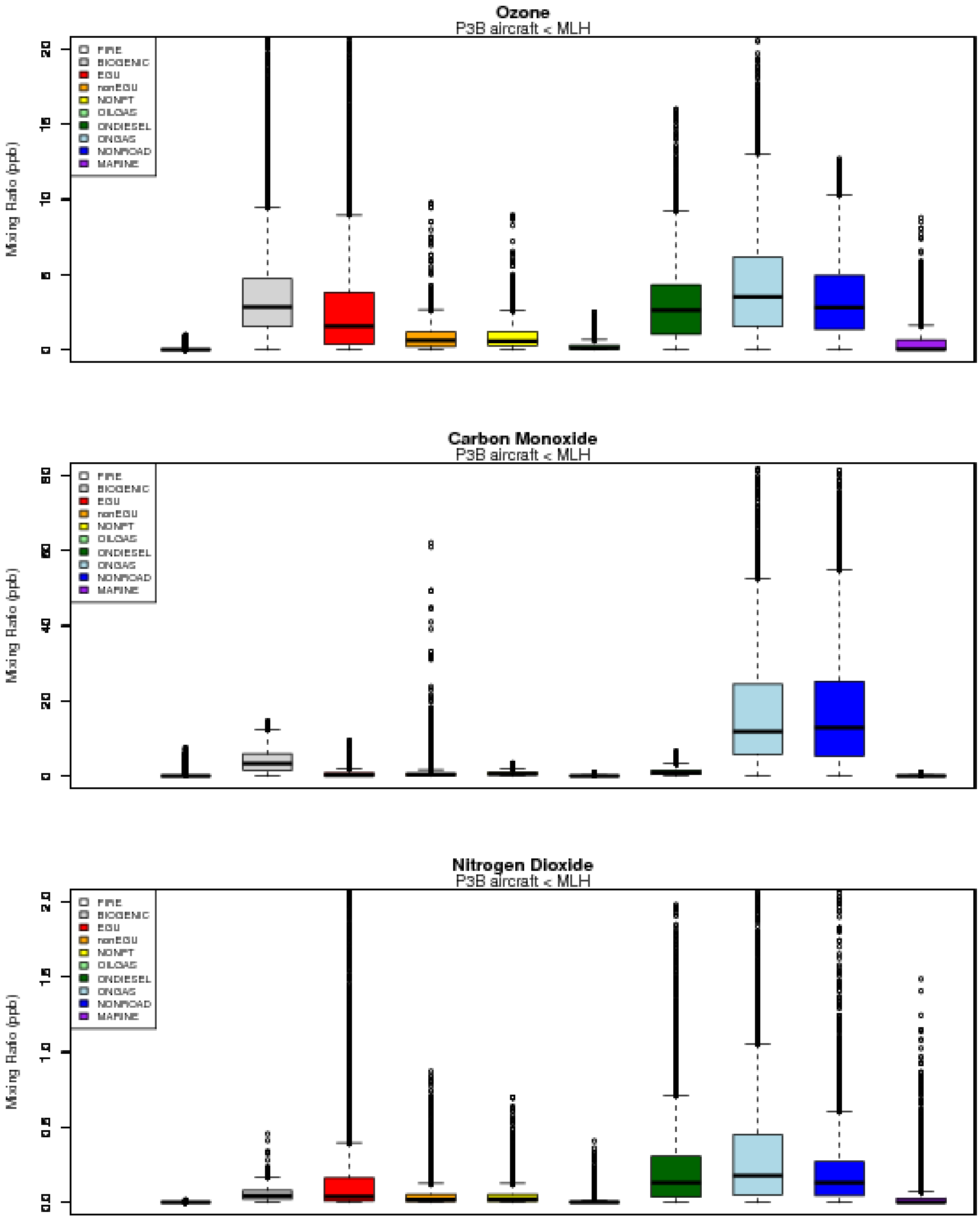

Figure S18: Distribution of modeled O3 (top), CO (middle) and NO2 (bottom) mixing ratio contributions within the boundary layer from each source category.

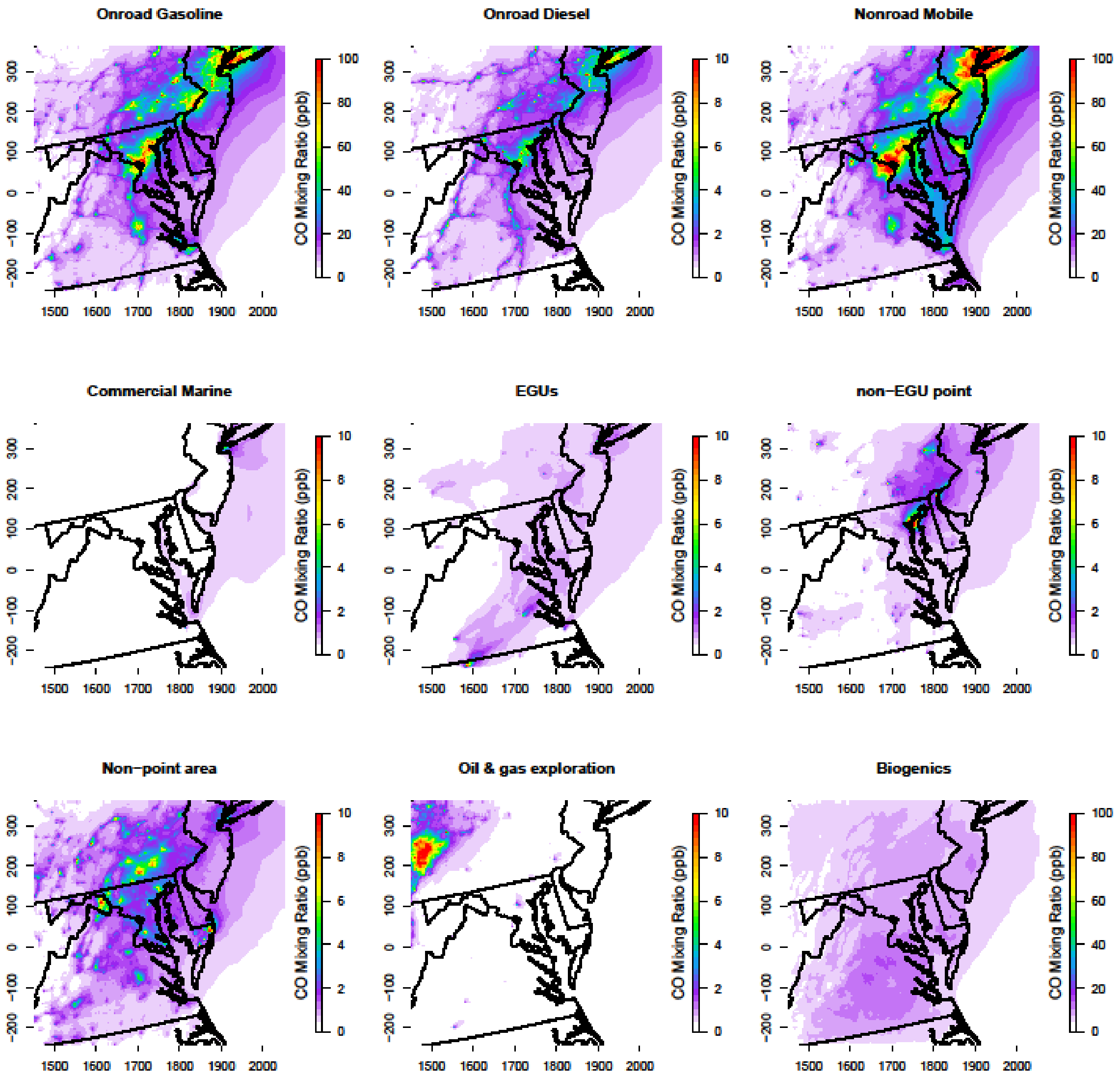

Figure S19: Average July 2011 spatial plots of contributions to CO from different source tags.

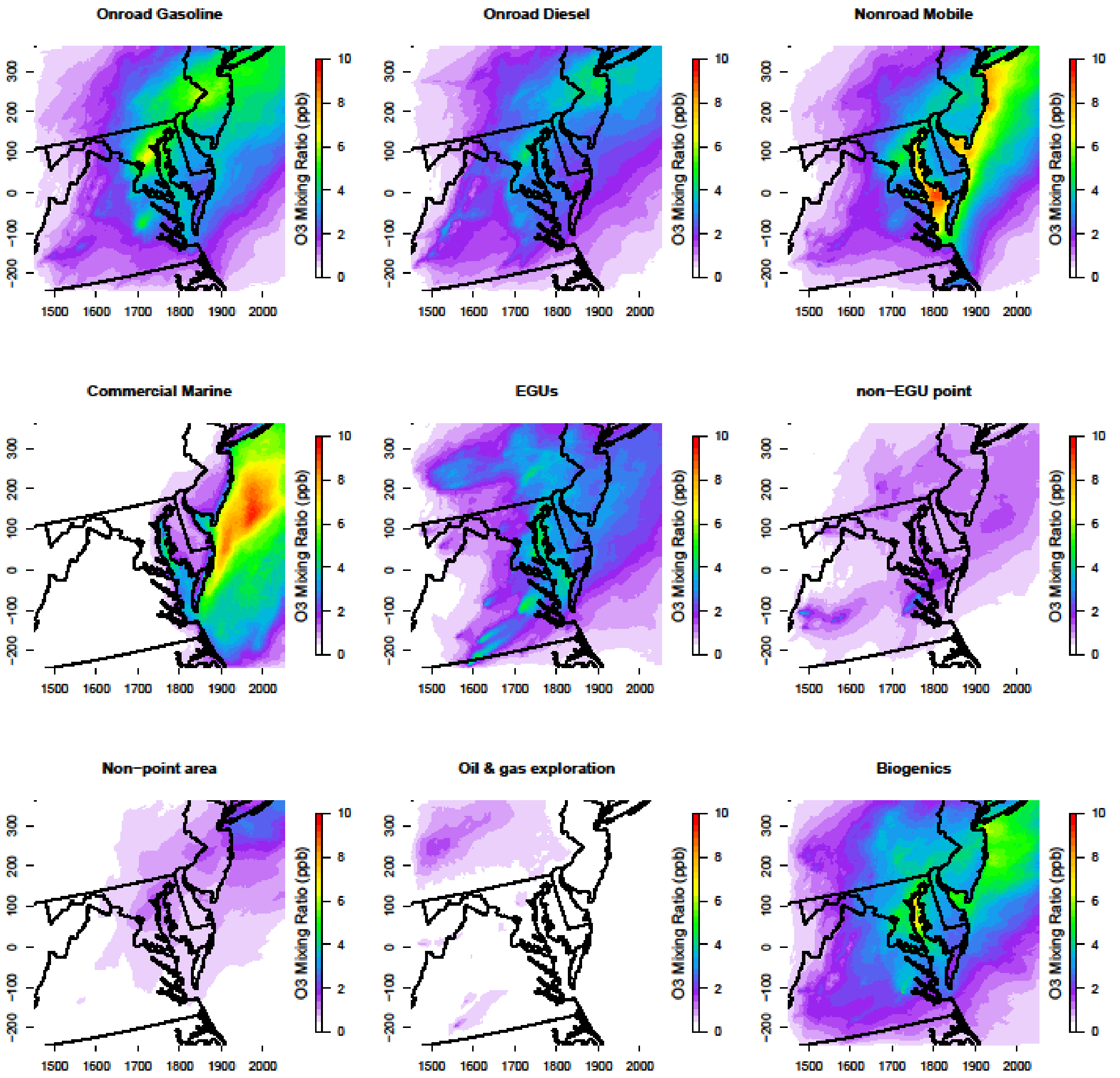

Figures S20: Average July 2011 spatial plots of contributions to ozone from different source tags.

Section S5: ΔCO:ΔNOy ratios from all ambient and modeled regressions

Table S2. ΔCO:ΔNOy ratios calculated using measured NOy (navy), ∑NOy,I (blue), and modeled NOy (pink) for each flight day in July and each location identified in Figure 1 for data collected in the boundary layer. Values derived from regressions that had Insignificant slopes are not shown. NA indicates no data available for regression on this flight day for this location. Gray shading highlights flight days and locations for which at least one regression was unavailable or had an insignificant slope. Orange shading highlights flight days and locations for which the 95% confidence intervals (2 times standard error) for ΔCO:ΔNOy from NOy and ∑NOy,i did not overlap.

	Aldino	Beltsville	Chesapeake Bay	Edgewood	Essex	Fairhill	Highway	Onflight	Padonia
7/1	2.5<br>2.5<br>5.2	2.8<br>3.0<br>8.1	7.4<br>9.0<br>9.5	1.3<br>3.2<br>5.9	4.7<br>6.1<br>9.8	3.5<br>5.1<br>11.1	4.1<br>5.4<br>10.5	4.1<br>3.1<br>9.7	2.0<br>Insig<br>9.3
7/2	12.3<br>13.4<br>15.0	9.1<br>11.4<br>8.7	10.0<br>Insig<br>15.4	9.1<br>10.2<br>10.9	3.9<br>3.2<br>5.6	10.3<br>9.7<br>12.0	5.0<br>10.4<br>8.2	7.5<br>8.9<br>10.9	8.3<br>6.9<br>13.3
7/5	2.3<br>3.8<br>10.5	4.4<br>5.7<br>6.7	11.0<br>23.0<br>Insig	5.1<br>8.0<br>9.1	4.9<br>7.2<br>8.9	5.4<br>6.2<br>8.6	4.6<br>4.6<br>10.1	4.7<br>6.3<br>7.5	4.5<br>NA<br>9.2
7/10	33.1<br>20.7<br>13.4	39.0<br>12.9<br>14.5	Insig<br>3.8<br>13.9	20.9<br>18.0<br>12.5	26.1<br>14.0<br>14.6	15.8<br>17.5<br>11.0	6.9<br>4.9<br>7.3	13.9<br>8.7<br>12.6	20.4<br>13.6<br>12.6
7/11	38.4<br>14.5<br>15.2	17.3<br>Insig<br>11.2	Insig<br>Insig<br>Insig	26.8<br>14.1<br>13.0	23.9<br>Insig<br>15.6	32.1<br>19.5<br>17.5	10.0<br>Insig<br>9.9	18.8<br>6.7<br>13.8	38.1<br>19.8<br>26.4
7/14	12.7<br>6.5<br>10.9	7.2<br>4.4<br>9.2	21.3<br>Insig<br>-12.5	10.8<br>6.7<br>11.8	10.0<br>4.9<br>9.1	12.1<br>8.3<br>10.6	3.4<br>2.8<br>10.2	4.7<br>4.8<br>10.4	8.9<br>Insig<br>11.5
7/16	11.9<br>11.9<br>10.4	7.6<br>8.1<br>10.9	Insig<br>Insig<br>14.7	7.8<br>8.5<br>8.7	18.4<br>14.8<br>8.7	14.7<br>14.2<br>9.4	5.1<br>8.3<br>9.3	7.3<br>9.2<br>11.7	11.2<br>10.6<br>13.8
7/20	6.7<br>5.7<br>10.3	5.9<br>8.8<br>12.8	30.3<br>22.3<br>3.8	14.7<br>16.0<br>2.8	7.4<br>8.3<br>9.3	11.7<br>13.7<br>13.3	2.6<br>3.8<br>11.4	5.9<br>4.5<br>9.1	13.2<br>11.0<br>13.7
7/21	17.9<br>16.3<br>9.6	9.7<br>9.8<br>Insig	NA<br>NA<br>NA	4.8<br>Insig<br>5.7	12.2<br>7.9<br>5.5	21.6<br>10.1<br>8.6	9.0<br>6.2<br>4.0	7.7<br>9.0<br>6.4	21.2<br>20.9<br>12.9
7/22	25.4<br>19.9<br>17.6	7.8<br>6.5<br>Insig	9.0<br>8.5<br>3.6	5.1<br>2.7<br>7.6	24.9<br>22.9<br>13.9	28.0<br>21.0<br>7.1	7.3<br>5.6<br>6.3	5.6<br>2.2<br>4.7	24.2<br>23.1<br>17.5
7/26	15.7<br>20.3<br>10.4	3.0<br>Insig<br>10.5	3.47<br>2.1<br>10.0	8.3<br>6.5<br>10.7	7.2<br>6.1<br>10.3	11.2<br>8.6<br>7.2	4.7<br>3.2<br>10.2	3.5<br>1.5<br>9.1	10.0<br>11.7<br>5.8
7/27	12.1<br>7.4<br>10.0	8.7<br>Insig<br>9.8	NA<br>NA<br>NA	9.1<br>Insig<br>6.4	7.9<br>Insig<br>7.4	12.3<br>7.9<br>11.6	3.5<br>Insig<br>10.2	10.6<br>10.6<br>7.6	9.2<br>Insig<br>4.6
7/28	17.5<br>Insig<br>10.3	3.7<br>Insig<br>12.7	-43.1<br>NA<br>13.1	7.5<br>Insig<br>6.8	8.3<br>NA<br>12.3	13.5<br>15.7<br>8.3	4.3<br>4.5<br>7.6	5.6<br>Insig<br>10.5	8.2<br>Insig<br>12.3
7/29	27.2<br>21.9<br>14.3	11.5<br>13.1<br>7.9	43.6<br>15.8<br>12.8	23.5<br>12.0<br>11.2	16.9<br>10.4<br>6.3	30.9<br>18.3<br>12.5	12.1<br>3.6<br>2.1	11.5<br>9.1<br>12.7	28.6<br>16.9<br>9.4

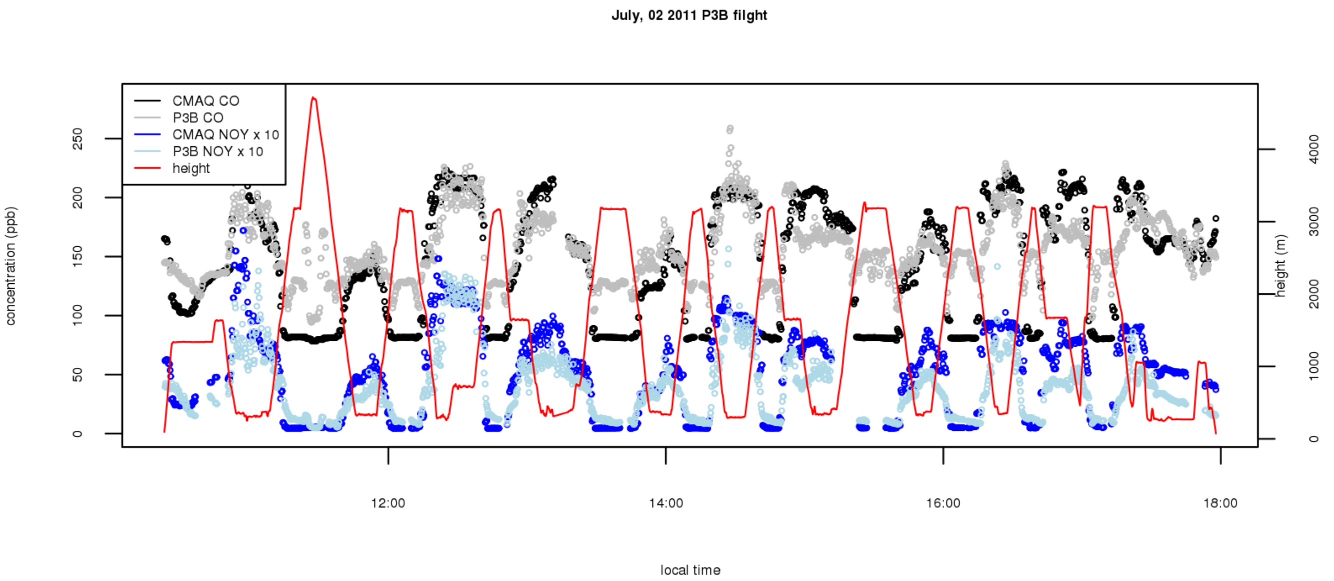

Figure S21: Time series of observed and modeled CO and NO_y matched in space and time along the aircraft flight track for July 2, 2011. Aircraft altitude is shown in red on the secondary y-axis.

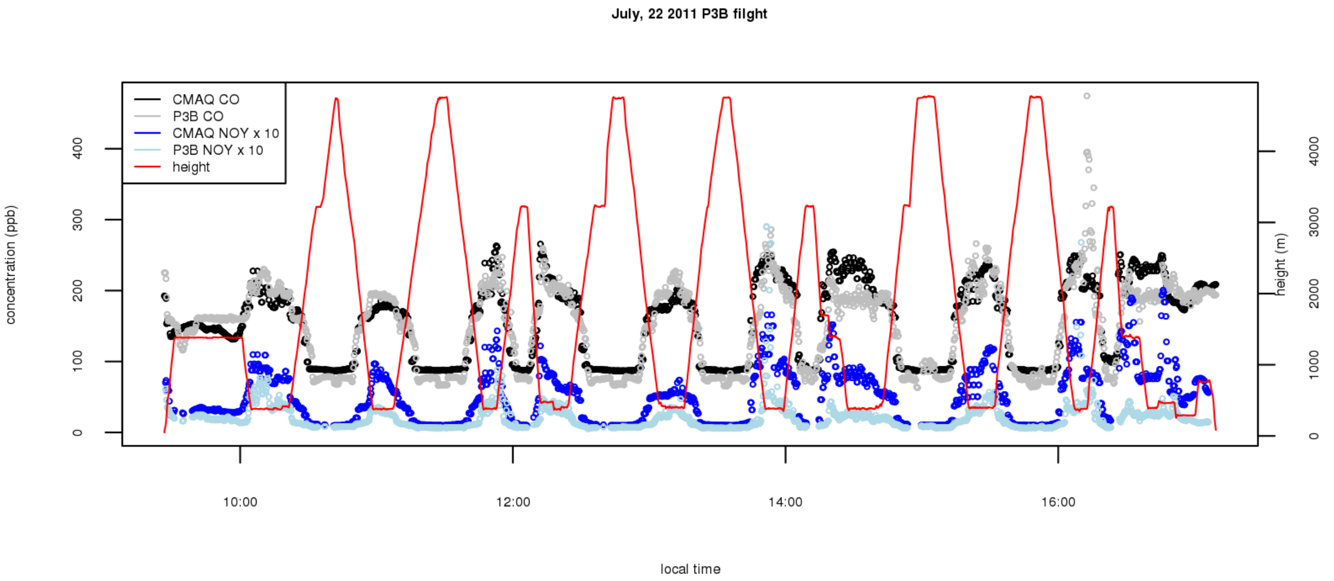

Figure S22: Time series of observed and modeled CO and NO_y matched in space and time along the aircraft flight track for July 22, 2011. Aircraft altitude is shown in red on the secondary y-axis.

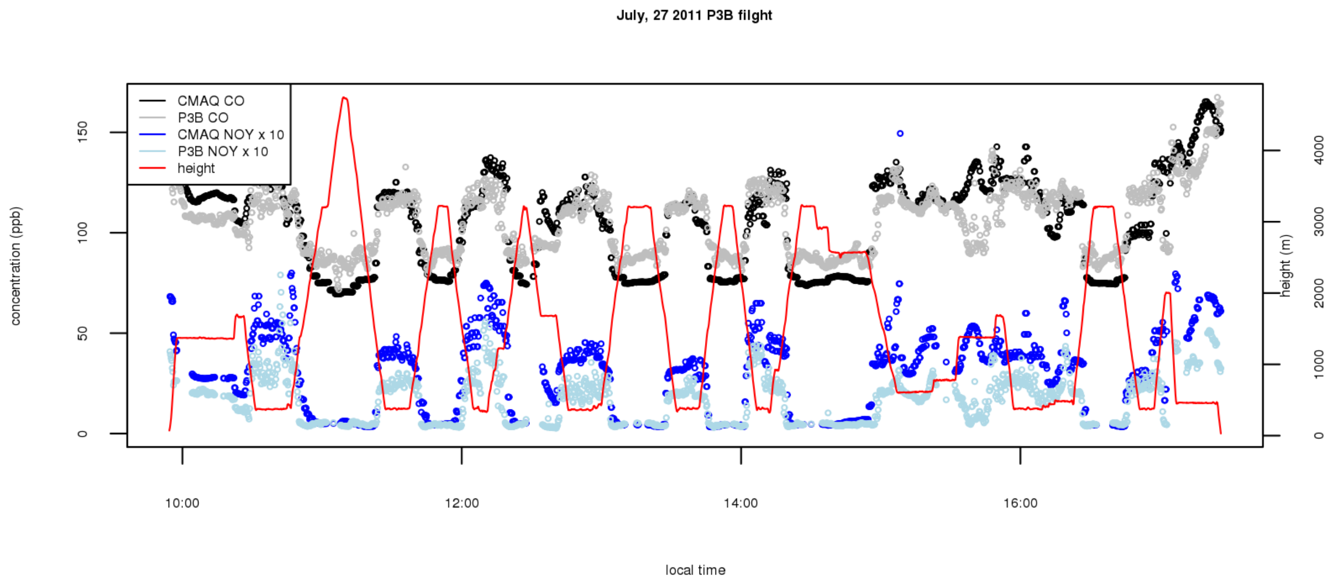

Figure S23: ime series of observed and modeled CO and NO_y matched in space and time along the aircraft flight track for July 27, 2011. Aircraft altitude is shown in red on the secondary y-axis.

Section S6. Modeled CO:NOy by source tag

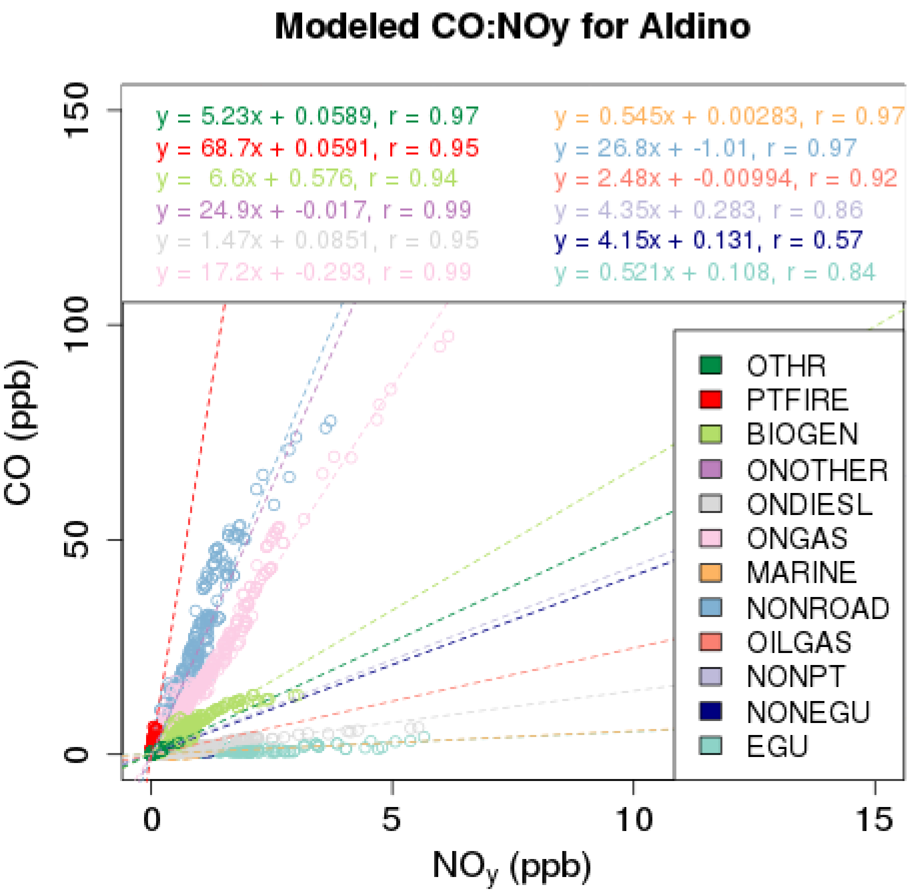

Figure S24: CO:NO$_y$ regressions for specific modeled source categories for Aldino. Source tag abbreviations defined in Table 1.
